# Over 40 Years of Fosmidomycin Drug Research: A Comprehensive Review and Future Opportunities

**DOI:** 10.3390/ph15121553

**Published:** 2022-12-14

**Authors:** Talea Knak, Mona A. Abdullaziz, Stefan Höfmann, Leandro A. Alves Avelar, Saskia Klein, Matthew Martin, Markus Fischer, Nobutada Tanaka, Thomas Kurz

**Affiliations:** 1 Institut für Pharmazeutische und Medizinische Chemie, Heinrich Heine University Düsseldorf, Universitätsstraße 1, 40225 Düsseldorf, Germany; 2 Initiative for Science & Society, Duke University, 140 Science Drive, Durham, NC 27708, USA; 3 Institut für Pharmazie, Universität Hamburg, Bundesstrasse 45, 20146 Hamburg, Germany; 4 School of Pharmacy, Kitasato University, 5-9-1 Shirokane, Minato-ku, Tokyo 108-8641, Japan

**Keywords:** DXR/IspC inhibitor, fosmidomycin, malaria, *Plasmodium falciparum*, *Mycobacterium tuberculosis*, PfDXR

## Abstract

To address the continued rise of multi-drug-resistant microorganisms, the development of novel drugs with new modes of action is urgently required. While humans biosynthesize the essential isoprenoid precursors isopentenyl diphosphate (IPP) and dimethylallyl diphosphate (DMAPP) via the established mevalonate pathway, pathogenic protozoa and certain pathogenic eubacteria use the less well-known methylerythritol phosphate pathway for this purpose. Important pathogens using the MEP pathway are, for example, *Plasmodium falciparum*, *Mycobacterium tuberculosis*, *Pseudomonas aeruginosa* and *Escherichia coli*. The enzymes of that pathway are targets for antiinfective drugs that are exempt from target-related toxicity. 2*C*-Methyl-D-erythritol 4-phosphate (MEP), the second enzyme of the non-mevalonate pathway, has been established as the molecular target of fosmidomycin, an antibiotic that has so far failed to be approved as an anti-infective drug. This review describes the development and anti-infective properties of a wide range of fosmidomycin derivatives synthesized over the last four decades. Here we discuss the DXR inhibitor pharmacophore, which comprises a metal-binding group, a phosphate or phosphonate moiety and a connecting linker. Furthermore, non-fosmidomycin-based DXRi, bisubstrate inhibitors and several prodrug concepts are described. A comprehensive structure–activity relationship (SAR) of nearly all inhibitor types is presented and some novel opportunities for further drug development of DXR inhibitors are discussed.

## 1. Introduction

The rapid spread of multi-drug-resistant (MDR) strains of pathogenic bacteria and parasites poses a global threat to human health. Thus, new drugs addressing unique therapeutic targets are urgently needed. Since its discovery in the early 1990s [1] by Rohmer et al., the 2*C*-methyl-D-erythritol 4-phosphate (MEP) pathway is accepted as an attractive, and for the treatment of malaria validated, target for the development of new anti-infective drugs. The MEP pathway is essential in several clinically relevant pathogens such as *Mycobacterium tuberculosis*, *Escherichia coli* (*E. coli*) and apicomplexan parasites including *Plasmodium* spp., and *Toxoplasma* spp., but is absent in mammals, fungi, archaebacteria and most Gram-positive bacteria such as *Streptococci* and some *Staphylococci* [1,2,3,4,5,6]. Over the course of seven enzymatic reactions, the MEP pathway leads to isopentenyl diphosphate (IPP) and dimethylallyl diphosphate (DMAPP), precursors to the isoprenoids. Since the enzymes of the MEP pathway have no human orthologs, target-related toxicity is not to be expected [7,8]. However, no MEP inhibitor has so far been approved as an anti-infective drug.

**Figure 1:**
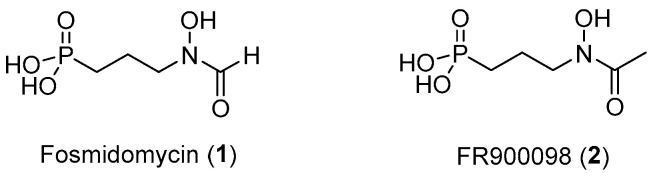
Structural formula of the natural product fosmidomycin (**1**) and its acetyl analog FR900098 (**2**).

Fosmidomycin (**1**) and FR9000098 (**2**) were first described in 1978 as antibiotics and herbicides (Figure 1) [9,10,11]. Twenty years later, they were identified as inhibitors of 1-deoxy-D-xylulose-5-phosphate reductoisomerase (DXR), the second and rate-limiting enzyme of the MEP pathway [12,13,14].

The phosphono-hydroxamic acids **1** and **2** possess potent antibacterial and antiparasitic properties. Unfortunately, the main shortcoming of both lead structures **1** and **2** are their unfavorable pharmacokinetic properties, mainly insufficient membrane permeability due to the charged phosphonate and the polar hydroxamate moiety and the short half-life time [15,16].

Over more than 40 years, significant efforts to improve the anti-infective properties have led to hundreds of novel DXR inhibitors based on the lead structures fosmidomycin (**1**) and FR900098 (**2**). In this review, we outline the physicochemical, pharmacokinetic and anti-infective properties of the parent compounds **1** and **2** as well as their efficacy spectra against various bacterial and parasitic pathogens. A short section of this review is dedicated to recent results of fosmidomycin (**1**) in human clinical trials. In addition, an overview of both the historical and recent development of novel DXR inhibitors with a particular focus on their activity against various pathogenic organisms and their structure–activity relationships (SARs) are also included. 

## 2. Discovery and Evaluation of Fosmidomycin (1) and Related Natural Products

**Figure 2:**
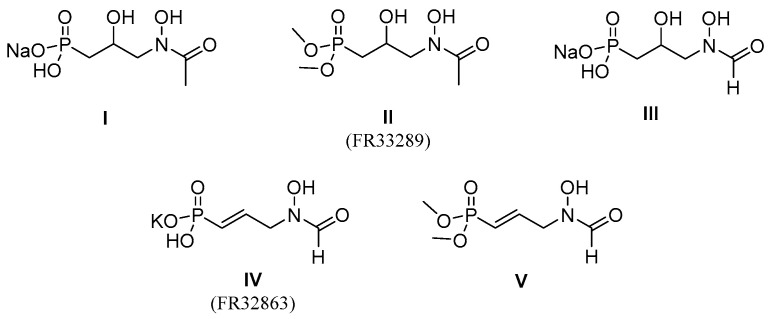
Additional phosphono-hydroxamic acids published by the Fujisawa Pharmaceutical Co., Ltd. [21].

In 1978, fosmidomycin (**1**, FR-31564) and FR900098 (**2**) were first described as a new class of antibiotics isolated from *Streptomyces lavendulae* and *Streptomyces rubellomurinus* [9,17]. The biosynthesis of FR900098 (**2**) has been completely elucidated, whereas, for fosmidomycin (**1**), only the biosynthesis of the putative precursor FR32863 (**IV**, Figure 2) is known [18,19,20]. Over the past four decades, the antiparasitic and antibacterial activities of **1** and **2** were determined, analyzed, and improved by various research groups. Alongside **1** and **2**, additional phosphono-hydroxamic acids were discovered by the Fujisawa Pharmaceutical Co., Ltd. (Figure 2) [21].

**Scheme 1:**
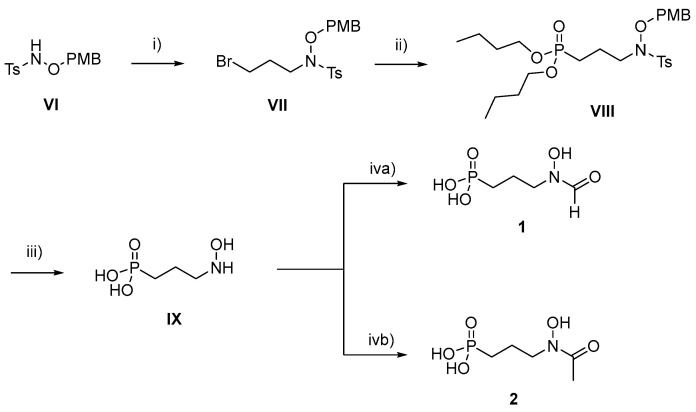
(**i**) 1. Na, EtOH, 70 °C, 1.5 h, 2. 1,3-dibromopropane, 2 h, rt to reflux, (**ii**) 1. NaH, benzene, dibutyl phosphonate, reflux, 3.5 h, 2. **VII**, reflux, 5.6 h (**iii**) 6 N HCl, acetic acid, 20 h, reflux (**iva**) 1. formic acid, acetic anhydride, 1.1 h, 0 to 5 °C, 2. NH_3_ (28 % aq. sol.), (**ivb**) acetic anhydride, water, rt, 1.5 h, Ts = tosyl, PMB = p-methoxybenzyl.

In a 1978 patent, Fujisawa Pharmaceutical Co., Ltd. described the first synthesis of both **1** and **2** as shown in Scheme 1 [10]. The four-step synthesis consists of the alkylation of an *N,O*-diprotected hydroxylamine (**VI**) with 1,3-dibromopropane, followed by a Michaelis-Becker reaction of propyl bromide **VII** with dibutyl phosphonate to give the protected phosphonic ester (**VIII**). After the removal of the protecting groups, the free hydroxylamine (**IX**) was formylated and acetylated producing the natural compounds (**1**) and (**2**). 

In 1989, Shigi identified fosmidomycin as an antibiotic inhibiting isoprenoid biosynthesis [22]. Towards the end of the millennium, in 1998, fosmidomycin (**1**) and FR900098 (**2**) were identified as selective inhibitors of *Ec*DXR by Kuzuyama et al. [12]. Additionally, in a Science publication in 1999 Jomaa and coworkers described fosmidomycin (**1**) as a *Pf*DXR inhibitor, inhibiting the growth of *P. falciparum* and also possessing curative properties in mice infected with *Plasmodium vinckei* [13]. In 2002, Kremsner and coworkers conducted a small-scale clinical trial of fosmidomycin for the treatment of uncomplicated malaria in adults, laying the foundation for further clinical trials [23].

### 2.1. Anti-Infective Activity of Fosmidomycin

Fosmidomycin inhibits a broad spectrum of pathogens which rely on the MEP pathway for the biosynthesis of DMAPP IPP. These pathogens include the apicomplexans *P. falciparum* (*Pf*) and *Toxoplasma gondii* (*Tg*) as well as bacteria [24]. The majority of pathogens use the MEP pathway, which includes Gram-negative bacteria such as *E. coli* (*Ec*) [25], *Acinetobacter baumannii* [26], *Klebsiella pneumonia* [26], *Pseudomonas aeruginosa* [27] and Gram-positive bacteria, including certain species of *Staphylococcus* [28]. Some bacteria use both, the MEP and the mevalonate pathway, for the synthesis of DMAPP and IPP, with the mevalonate pathway being a more recent addition to some species [29]. Pathogens that feature both pathways include *Listeria monocytogenes* and some species of *Streptomyces* [28]. In contrast, examples of pathogenic bacteria that exclusively use the mevalonate pathway includes Gram-positive genus of *Streptococcus* and the Gram-negative genus of *Borrelia* [28,30]. The application of fosmidomycin is limited to species that solely rely on the non-mevalonate pathway for isoprenoid synthesis. 

To date, the DXRs of *P. falciparum*, *E. coli* and *M. tuberculosis* (*Mt*) are the best-studied enzymes. As a result, most synthesized fosmidomycin analogs have been tested against at least one of these enzymes, in addition to cellular assays. Additional information about the efficacies of **1** and **2** against these pathogens can be found in the Supplementary Materials (Table S1).

Although the enzyme catalytic sites are highly conserved across pathogens [31,32], whole-cell activities for inhibitors differ greatly due to significant distinctions among the organisms as a whole, including different localization of the DXR enzymes. In bacteria, the enzyme resides in the cytosol, whereas the homologs in parasites are located in the apicoplast, a plastid-like cell organelle accommodating a variety of biosynthetic pathways [24]. Uptake mechanisms of fosmidomycin also differ among pathogens. In *E. coli*, a cAMP-dependent glycerol 3-phosphate transporter facilitates an effective drug uptake resulting in bacterial death. 

In contrast, *M. tuberculosis* lacks a similar transporter, making the bacteria intrinsically resistant to fosmidomycin as the inhibitor cannot penetrate the cell wall via passive diffusion [33]. Additionally, the cell wall of *Mt* contains highly lipophilic mycolic acids, which prevent cell wall penetration of polar drugs, including fosmidomycin, in typically used concentrations [33]. For human erythrocytes infected with *Pf*, a parasite-induced pathway known as the new permeability pathway was proposed to be most likely responsible for drug uptake [15]. Treatment of *Pf* with fosmidomycin resulted in reduced amounts of MEP pathways metabolites and their resulting isoprenoids [34]. Parasite growth is inhibited in the first cell cycle after haemoglobin digestion and DNA replication has been initiated [35].

#### 2.1.1. Parasites

**Figure 3:**
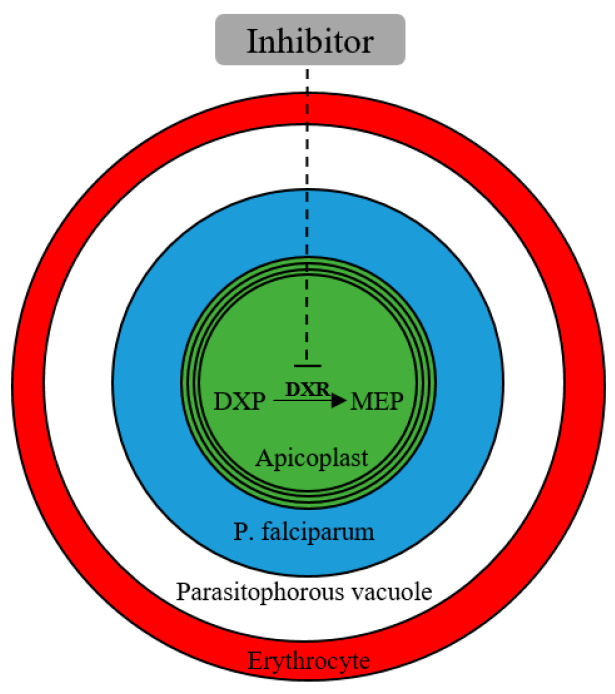
Passage of DXR inhibitors across seven membranes in *Plasmodium*-infected erythrocytes. Adapted with permission from *J. Med. Chem*. 2015, 58, 4, 2025–2035 [37]. Copyright 2022 American Chemical Society.

In vitro and in vivo, the *Pf* parasites infect human erythrocytes for asexual reproduction. Besides the erythrocyte membrane, the parasitophorous vacuole membrane (PVM) and the plasmodium cell membrane must also be overcome. Inside the *Plasmodium* parasite, DXR is localized in the apicoplast which contains additional four membrane layers (Figure 3) [36].

Fosmidomycin is able to kill *Pf* pathogens (IC_50_ = 0.81 µM) [37], but not *Toxoplasma gondii.* In both pathogens, the DXR enzyme is located in the Apicoplast. Nevertheless, fosmidomycin seems to be unable to penetrate the membranes of *T. gondii* while penetration through *Pf* membranes is possible [15]. A likely cause for fosmidomycin’s inability to act upon *T. gondii* is the lack of the glycerol-3-phosphate transporter (*GlpT*), which is known to be responsible for fosmidomycin uptake in *E. coli* and other pathogens [34]. Interestingly, strains of *T. gondii* engineered to express GlpT are susceptible to fosmidomycin. Enzyme assays performed on *T. gondii* DXR have shown, that both fosmidomycin (K_i_ = 90 nM) and FR900098 (K_i_ = 48 nM) are potent inhibitors [34], paving the way for fosmidomycin-based treatments if the permeability issues can be overcome by structural modification [15].

*Babesia orientalis* is a tick-borne apicomplexan parasite and the cause of water buffalo babesiosis. While humans are not affected by this pathogen, its eradication is of interest as it causes considerable economic loss, especially in China. Fosmidomycin was able to limit the growth of *B. orientalis*, with the treatment of the pathogen leading to a significant reduction in relative growth [38]. Similar results were reported in *B. bigemina* and *B. bovis*, with clearance achievable in 3 days and 4 days, respectively. Both parasite species were incapable of growth after in vitro treatment with fosmidomycin, suggesting that fosmidomycin may be an effective drug for the treatment of bovine babesiosis [39].

*Eimeria tenella* causes eimeriosis in poultry and poses a major threat to food security. Impairment of parasitic growth required higher concentrations of fosmidomycin compared to *P. falciparum* to be statistically significant [40]. The poor efficacy of fosmidomycin was attributed to different factors, including inactivation of the active drug, poor permeability, and/or efflux of the drug. Taking similar results from *T. gondii* and the absence of the MEP pathway in the *Cryptosporidium* genus altogether into consideration these findings imply heterogeneity among apicomplexan parasites [40].

#### 2.1.2. Gram-Positive Bacteria

The *Staphylococcus* genus is unique in that it features species that rely on either pathway for isoprenoid synthesis. Fosmidomycin inhibited the growth of *S. schleiferi* (MIC = 0.5–8 µg/mL) and *S. pseudintermedius* (MIC = 0.5–1 µg/mL) which are associated with household animal infections and both possess all enzymes of the non-mevalonate pathway. However, fosmidomycin could not cure infections with *S. aureus*, *S. epidermidis* and *S. lugdenensis,* which lack the *dxr* gene [28,41]. These findings contradicted earlier reports of fosmidomycin and FR900098 having shown activity against *S. aureus* [42]. A recent publication by Edwards et al. showed that fosmidomycin is indeed inactive against *S. aureus*. Edwards et al. also laid out a resistance mechanism towards fosmidomycin in *S. schleiferi* and *S. peudintermedius*, mediated by mutations lowering the function of GlpT and leading to decreased drug uptake into the aforementioned pathogens [43].

#### 2.1.3. Gram-Negative Bacteria

The Gram-negative bacterium *E. coli* is often considered to be a model organism for anti-bacterial drug research, but a survey conducted on clinical isolates in 2018 showed that 58% of samples were resistant to current treatment options [44]. Fosmidomycin is a moderate agent against the K12 strain of *E. coli* (MIC = 12.5 µM) [45]. While fosmidomycin showed potent enzyme inhibitory activity against the wild type of *Ec*DXR (IC_50_ = 0.03 µM), several mutations have been observed that decreased activity by up to 10-fold [25]. A fosmidomycin resistance gene (*fsr*) was also originally discovered in *Ec*, most likely encoding for an efflux pump that increased resistance by more than 30-fold. This efflux pump seems to be specific for fosmidomycin and does not act upon other antibiotics apart from trimethoprim [46,47]. It could be shown that *E. coli* can grow even after the deletion of the genes encoding for DXS and DXR. Rodríguez-Concepción and coworkers described that in the case of *dxs* deletion, mutations in the *ribB* and *aceE* genes lead to enzymes capable of supplying DXP. A mechanism for survival of DXR deletion has yet to be postulated and is of great interest to elucidate a new possible way of fosmidomycin resistance [48,49].

Strains of the *Burkholderia* genus, pathogens related to opportunistic infections of the respiratory tract in cystic fibrosis patients, were mostly resistant to both fosmidomycin and FR900098 as well as other conventional antibiotics [50]. Resistance was mostly attributed to insufficient retention of inhibitors within bacterial cells, caused by the upregulation of *fsr*. This resistance could be partially circumvented by the addition of glucose-6-phosphate to the medium, prompting an increase of genes related to glycerol-3-phosphate uptake into bacterial cells, thus facilitating FR900098 (**2**) uptake [50]. A combination of fosmidomycin and colistin reduced the MIC of colistin by up to 64-fold in clinical isolates of *B. multivorans*, an effect that could be attributed to increased membrane permeability [51].

*Francisella tularensis* is a Gram-negative bacterium and the cause of tularemia, a zoonotic disease transmitted by rodents and lagomorphs [52]. Jawaid et al. showed that fosmidomycin reduced in vitro growth of *F. tularensis* subspecies *novicida* by inhibition of *F. tularensis* DXR (MIC = 136 µM) [53]. Clinical isolates of *Francisella* were resistant to *β*-lactam antibiotics due to the expression of *β*-lactamases and spontaneously occurring resistance to fosmidomycin has also been described. Similar to *S. schleiferi* and *S. pseudintermedius*, this resistance was mediated by mutations in the GlpT gene [52].

The causative agent of the plague, *Yersinia pestis*, garnered attention over its potential applications for bioterrorism [54] and the 2017 plague outbreak in Madagascar [55]. The disease mostly manifests in two forms: the bubonic and pneumonic plague [55]. Both fosmidomycin (IC_50_ = 0.71 µM) and FR900098 (IC_50_ = 0.23 µM) showed submicromolar inhibitory activity [56]. Both agents lacked the ability to inhibit the growth of *Y. pestis*, even though uptake of fosmidomycin was likely mediated by a transport protein homologous to the *E. coli* GlpT transporter [57].

*Acinetobacter baumannii* is a Gram-negative bacterium and the cause of a plethora of nosocomial infections, including soft-tissue infections, pneumonia, septicemia, and urinary tract infections [58]. Treatment of emerging multidrug-resistant *A. baumannii* infections often requires reserve antibiotics such as carbapenems in combination with colistin or an aminoglycoside. Fosmidomycin (IC_50_ = 47 nM) and FR900098 (IC_50_ = 24 nM) both exhibited nanomolar activity against *Ab*DXR but only FR900098 showed activity against selected *A. baumannii* strains in a whole-cell assay [26]. Resistance to fosmidomycin and FR900098 in certain *A. baumannii* strains was theorized to be based on a lack of GlpT uptake or poor permeability.

The Gram-negative bacterium *Klebsiella pneumoniae* naturally resides on the skin as well as in the nasopharyngeal and intestinal tracts of both humans and mammals. *K. pneumoniae* is opportunistically pathogenic and a leading cause of nosocomial infections. The pathogen is not inherently resistant to antibiotics but is known for its ability to acquire multidrug resistance plastids [59]. Both fosmidomycin (IC_50_ = 20 nM) and FR900098 (IC_50_ = 23 nM) showed equal nanomolar activity in an enzyme assay, with fosmidomycin also exhibiting weak activity in a whole-cell assay (MIC = 64–128 mg/L). The superior activity of fosmidomycin over its acetyl derivate (MIC = 256 mg/L) may be attributed to a more facile uptake via the GlpT [26].

In addition to the above-listed pathogens, fosmidomycin and FR900098 have been tested against other bacteria listed in Table S1 of the Supplementary Materials. Information on those pathogens is limited, though noteworthy examples include *Bacillus anthracis* and *Pseudomonas aeruginosa*.

### 2.2. Pharmacokinetic Profile of Fosmidomycin

Fosmidomycin and FR900098 both contain a phosphonic acid group, which is connected via a propyl linker to *N*-formylated (**1**) or *N*-acetylated (**2**) hydroxylamine moieties, leading to highly polar, water-soluble and stable compounds. Due to its dianionic structure in a physiological medium, the phosphonate group (pK_a1_ = 2.2, pK_a2_ = 6.7) [60], is mainly responsible for the excellent aqueous solubility of both compounds. The high water solubility on the other hand results in unfavorable permeability [61,62], as well as a comparatively short plasma half-life of approximately 1.87 h due to rapid renal excretion [63]. The absorption half-life of fosmidomycin via a one-compartment model was determined at 0.4 to 1.1 h [64]. No metabolites of **1** are known and the active agent is excreted renally [65]. An advantageous trait of **1** and **2** is their low cytotoxicity as determined in a mouse model. In addition to these early findings more recent clinical trials have confirmed the generally low toxicity of fosmidomycin paving the way for further clinical trials in humans [66,67].

In vivo studies in humans best fit with a one-compartment model and first-order absorption and elimination of fosmidomycin [67]. Plasma protein binding is typically low for a hydrophilic therapeutic agent at about 1% [67]. No mutagenic potential has been reported for fosmidomycin, although the formation of an *N*-substituted hydroxylamine upon hydrolysis is theoretically possible [68]. Hydroxylamines have been reported to have mutagenic potential [69]. Fosmidomycin is typically administered two to four times per day with an upper daily dose of 3600 mg per day [67]. Expectedly, the fluctuation of fosmidomycin’s plasma concentrations is lower if smaller doses are administered more frequently compared to larger doses over a larger interval. More frequent applications of smaller doses also result in higher minimum plasma concentrations at a steady state. The mode of action of fosmidomycin seems to be time-dependent rather than concentration-dependent [64]. This finding suggests more frequent applications are required to maintain consistently high plasma concentrations of the drug.

### 2.3. Clinical Trials from 1985 to 2018

Since its discovery, fosmidomycin has been the subject of several clinical trials, both as a standalone therapeutic and in combination with other approved antimalarials or antibiotics. In 1985, fosmidomycin phase I and phase II clinical trials for the treatment of urinary tract infections were conducted [70,71]. However, the study was discontinued for unknown reasons. In their third edition of the guidelines of malaria treatment, the WHO classifies treatments with a cure rate of 90% as acceptable [72]. A 2015 meta-analysis by Fernandes et al. pooled the data of ten clinical trials studying fosmidomycin, of which six were pediatric studies and the remaining four were involving adults [73]. Trials employing **1** as a single therapeutic agent failed to produce acceptable cure rates by the WHO’s standards [73]. More recent trials are focused on fosmidomycin combinations, for example with the antibiotic clindamycin for which Wiesner et al. showed a synergistic effect [66]. While most studies involved this combination, an approach that made use of artesunate instead of clindamycin is also included [73]. The meta-analysis showed that the combination of fosmidomycin with a second antimalarial led to a cure rate of 85% (95% CI: 71–98%) on day 28 in children and 70% (95% CI: 40–100%) respectively in adults. **1** proved to be a safe antimalarial, with adverse events mainly limited to gastrointestinal disturbance [73]. However, isolated cases of haematological changes such as neutropenia have been reported by Borrmann et al. [74]. A temporary hiatus in the clinical evaluation of fosmidomycin may be attributed to a 2012 trial by Lanaspa et al. that only produced a 43% cure rate on day 28 (95% CI: 27–59%) for children under the age of three [75]. In 2018; Mombo-Ngoma et al. published the results of a Gambon-based study involving fosmidomycin in combination with piperaquine [76]. The aim of this phase II study was to demonstrate the efficacy, tolerability and safety of the combination as a treatment of *P. falciparum* infections in both children and adults. The cure rate on day 28 across all age groups was reported to be 83.8% (95% CI: 75.1–90.5%). In addition to adverse effects concerning the gastrointestinal and respiratory tract, two out of the 100 enrolled patients showed a prolonged QT interval of >500 msec [76]. None of the completed trials produced cure rates that can be considered acceptable by the WHO’s standards, although the cure rate of 85% in children as determined in the meta-analysis by Fernandes et al. comes close [73]. Because the dosage of fosmidomycin is already high, further increasing the dose for fosmidomycin alone may not be feasible and may not result in better cure rates. In vitro, a decrease in Vγ9/Vδ2 T cell response, which can detect (*E*)-4-hydroxy-3-methylbut-2-enyl pyrophosphate (HMB-PP) as a key intermediary of the MEP pathway, has been observed. The significance of this observation has not yet been assessed in vivo [77]. Fosmidomycin’s shortfall in efficacy underlines the necessity of either inclusion of an additional antimalarial into existing fosmidomycin-based combination therapies or the introduction of structural modifications to fosmidomycin to improve cure rates. 

As of February 2022, there are no ongoing fosmidomycin clinical trials listed on ClinicalTrial.gov, though the Deutsche Malaria GmbH recently announced a trial of triple therapy using fosmidomycin, clindamycin and artesunate. The trial will be supported by the EU Malaria Fund and aims to enroll more than 5000 patients, making it the largest single trial of fosmidomycin in humans [78]. No timeline or further updates regarding this study have been published.

## 3. 1 Targeting the Deoxy-D-xylulose-5-phosphate Reductoisomerase (DXR) 

**Figure 4:**
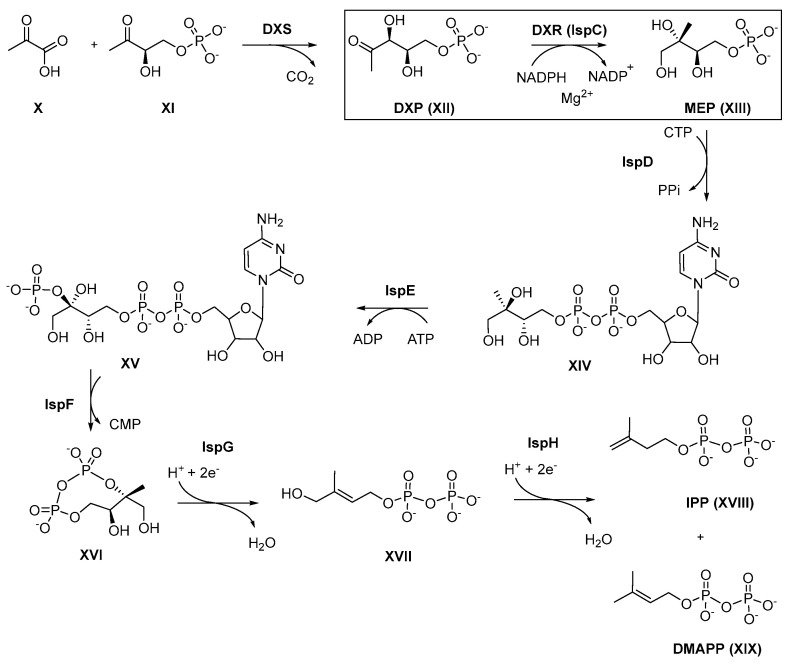
The MEP pathway leading to the isoprenoid precursors isopentenyl diphosphate (**XVII, IPP**) and dimethylallyl diphosphate (**XIX, DMAPP**) via an IspC/DXR-catalysed conversion of 1-deoxy-D-xylulose 5-phosphate (**XII, DXP**) to 2-C-methyl-D-erythritol 4-phosphate (**XIII, MEP**).

The MEP pathway begins with the synthesis of 1-deoxy-D-xylulose 5-phosphate (DXP) from two glycolytic intermediates, pyruvate (**X**) and glyceraldehyde 3-phosphate (**XI**) catalyzed by DXP synthase (DXS), concluding with the production of IPP and DMAPP after six catalytic steps (Figure 4) [79].

In the second step 1-deoxy-D-xylulose 5-phosphate reductoisomerase (DXR/IspC), a homodimer catalyzes the intramolecular rearrangement and reduction of DXP (**XII**) to MEP (**XIII**) [80]. The complex conversion of DXP to MEP requires the presence of the cofactor NADPH and a bivalent metal ion, e.g., Mg^2+^ or Mn^2+^ [81].

**Figure 5:**
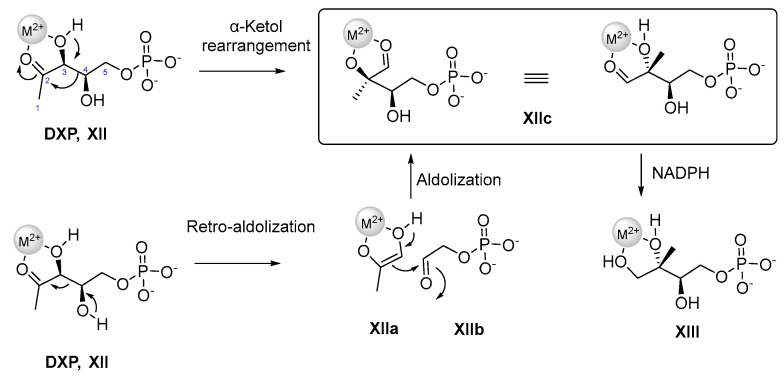
Two conceivable mechanisms for the enzymatic mode of action of DXR involving a divalent metal cation M^2+^ (grey sphere) and NADPH [87]. Used with permission of EUREKA SCIENCE, from Targeting the MethylErythritol Phosphate(MEP) Pathway for Novel Antimalarial, Antibacterial and Herbicidal Drug Discovery: Inhibition of 1-Deoxy-D-Xylulose-5-Phosphate Reductoisomerase (DXR) Enzyme, Nidhi Singh, Volume 13, Issue 11, 2007; permission conveyed through Copyright Clearance Center, Inc.

At least two possible reaction mechanisms (Figure 5) have been proposed for the DXR-catalyzed isomerization of **XII** to 2-C-methyl-D-erythrose 4-phosphate **(XII c)**. One reaction mechanism for the formation of intermediate **XIIc** is based on an *α*-ketol-rearrangement, in which the C3 hydroxyl group of **XII** is deprotonated, followed by a subsequent 1,2-alkyl shift. The C3-C4 carbon-carbon bond is cleaved in a way that C4 can thereafter attack the carbonyl C2. The 1-hydroxy 2-ethyl phosphate translocates to the C2 position, forming **XIIc** [82,83].

An alternative approach is a stepwise retro-aldol/aldol-mechanism [82,83,84]. In the retro-aldol step, the oxidation of the C4 carbon atom of **XII** causes a C-C bond break between C3 and C4, whereby a hydroxyacetone enolate **XIIa** and an aldehyde phosphate **XIIb** are formed as intermediates [85,86]. In the following aldolization step, the hydroxyacetone enolate **XIIa** attacks the aldehyde phosphate **XIIb**, forming **XIIc** in an electrophilic attack. A new bond is formed between the C2 carbon atom of the enolate and the C1 atom of the aldehyde phosphate **XIIb**. In the last step, **XIIc** is reduced to MEP **XIII** by NADPH.

MEP is the substrate of IspD, which catalyzes the reaction with cytidine 5′-triphosphate (CTP) to give methylerythritol cytidyl diphosphate (**XIV**). Subsequently, the C2 hydroxyl group of **XIV** is phosphorylated by IspE using ATP as a phosphate donor. The resulting phosphate ester 4-diphosphocytidyl-2-C-methyl-D-erythritol-2-phosphate (**XV**) is then cyclized by IspF to 2-C-methyl-D-erythritol-2,4-cyclodiphosphate (**XVI**). In the following step, IspG catalyzes the reductive dehydratisation and ring opening to yield 4-hydroxy-3-methyl-butenyl 1-diphosphate (**XVII**). Finally, the reductive dehydroxylation of **XVII** provides both isopentenyl diphosphate (IPP, **XVIII**) and dimethylallyl diphosphate (DMAPP, **XIX**) [79].

### 3.1. Crystal Structures of DXR

**Figure 6:**
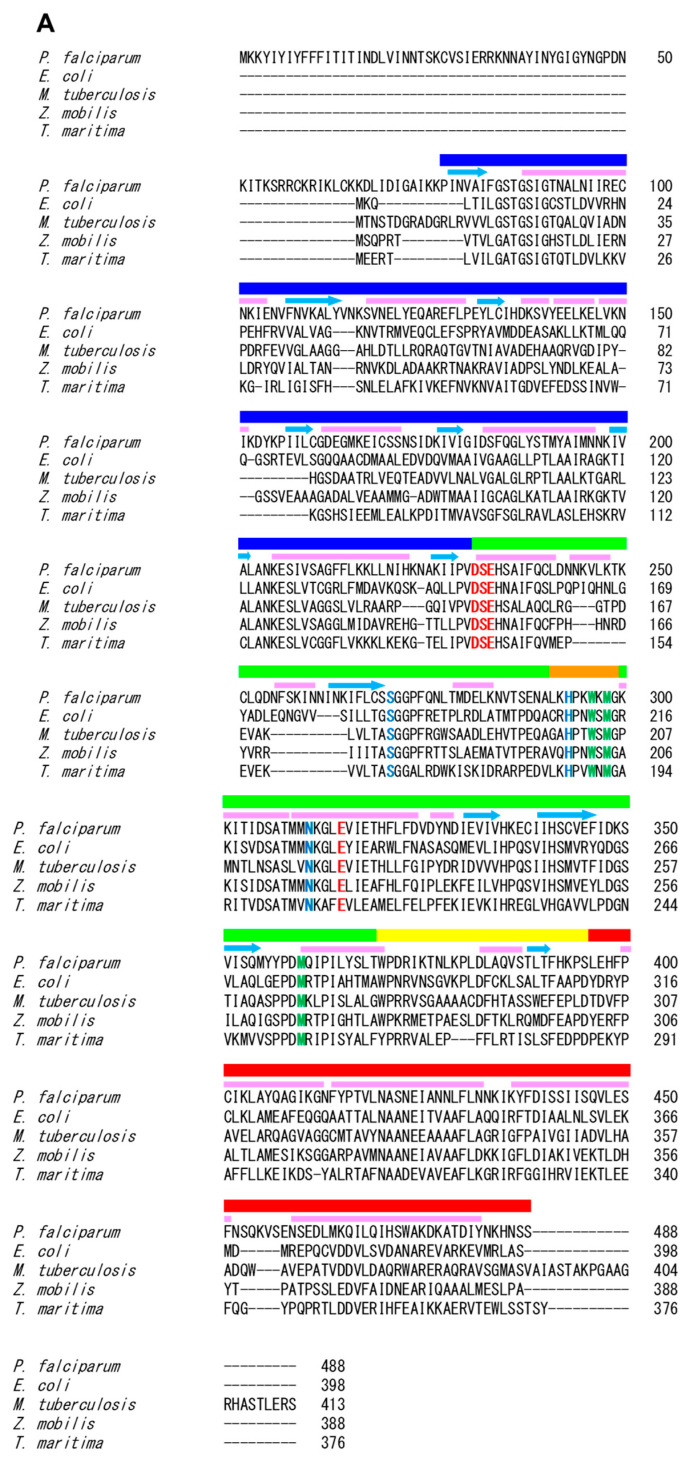

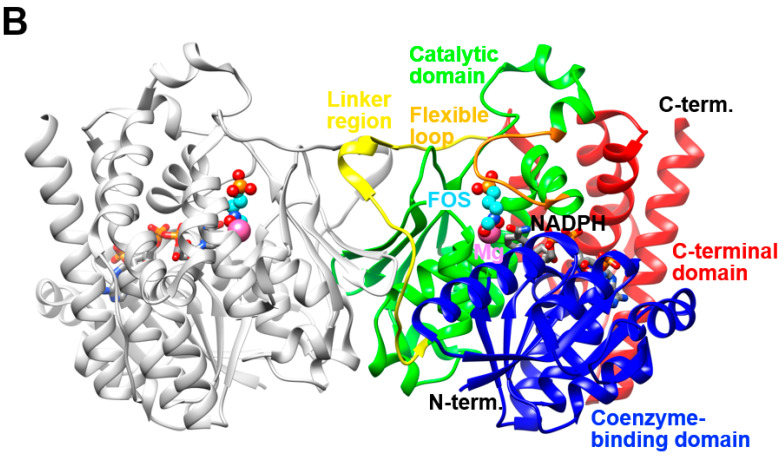
(**A**): Amino acid sequence alignment of bacterial and parasitic DXRs. Residues involved in phosphate/phosphonate-, linker-, and metal and hydroxamate-binding are highlighted in blue, green, and red, respectively. The colored ribbons above the sequence alignment represent the respective domains in *Pf*DXR: the NADPH-binding (blue), catalytic (green), and C-terminal (red) domains. The linker region and flexible loop in the catalytic domain are colored yellow and orange, respectively. The pink bars and cyan arrows represent the secondary structure elements, namely, α-helices and β-strands, respectively; (**B**): The overall structure of the quaternary (enzyme-NADPH-metal-inhibitor) complex of *Pf*DXR (PDB 3AU9) [91]. Three domains, a linker region, and a flexible loop in the catalytic domain of one subunit are colored as in (**A**). The other subunit is colored grey. The bound fosmidomycin (FOS) and NADPH molecules are shown as ball-and-stick (cyan) and stick (grey) models, respectively. The bound magnesium ions are shown as sphere models (pink).

1-Deoxy-D-xylulose 5-phosphate reductoisomerase (DXR) is present in more than 400-annotated (Swiss-Prot) entries in the Uniprot database. These entries consist primarily of bacteria with some examples of eucaryota. The length of the amino acid (aa) sequence varies from 356 aa in *Campylobacter jejuni* to 488 in *Plasmodium falciparum* (Figure 6A) [88]. Presently, the DXR structures of *E. coli* (*Ec*DXR), *P. falciparum* (*Pf*DXR), and *M. tuberculosis* (*Mt*DXR) have been more thoroughly characterized and studied [89,90].

DXRs are homodimers. The subunit of DXR consists of two large domains separated by a cleft containing a deep pocket, a linker region, and a C-terminal domain (Figure 6B). One of the large domains is the N-terminal NADPH binding domain and the other is the catalytic domain which provides the groups necessary for catalysis (metal and substrate binding). The N-terminal NADPH-binding domain is connected to a catalytic domain. The N-terminal domain (NTD) comprises the first 150 amino acids. The structural organization of the NTD resembles the Rossman fold, which is found in proteins showing interactions with dinucleotides. This region shows high similarity among orthologues *Ec*DXR, *Mt*DXR and *Pf*DXR.

The central catalytic domain comprises 125 amino acids and due to the metal-based mechanism of catalysis, acidic amino acids responsible for the binding of the divalent metal are found in this domain (Figure 6A). Another important characteristic of the substrate-binding site is the flexibility of the domains, a feature necessary for the complex enzymatic process involving both a divalent cation and NADPH. In DXR crystals, the relative position of the NADPH-binding and catalytic domains exhibits different conformations depending on the presence of co-crystallized ligands, cofactors, and substrates. These conformations are highly dependent on the position of a flexible loop located at the entrance of the substrate-binding site, causing the catalytic site to be in a closed, open, or super-open state [31,91,92]. The connection between the catalytic domain and the C-terminal domain is made via a sequence of around 30 amino acids known as the linker region. In the crystal structures of DXR, the linker region as well as a *β*-strand of the catalytic domain from each subunit are involved in the dimer interface. The C-terminal domain of DXR is formed by a four-helix bundle motif, showing a high degree of flexibility and no interface of contact between the dimer subunits [31,92,93].

An analysis of the similarity between the orthologues *Ec*DXR, *Mt*DXR, and *Pf*DXR using BLAST revealed that *Ec*DXR shares 40% amino acid sequence identity with *Mt*DXR and 37% with *Pf*DXR, while *Mt*DXR and *Pf*DXR share 34% identity [89]. Despite the sequence identity of the proteins ranging from 34% to 40%, the overall three-dimensional arrangement of the enzymes co-crystalized with both cofactors is similar [31]. The major part of the dissimilar regions occupies solvent-exposed areas.

The first *Ec*DXR crystal structure was independently determined in 2002 by the Stubbs group (PDB 1K5H) [31] and the Ohsawa group (PDB 1JVS) [94]. The structure of *Mt*DXR was first solved in 2006 [95] and *Pf*DXR in 2011 [91]. Since then, solid efforts by several groups led to the obtention of crystal structures of DXR from different organisms. Currently, 77 DXR crystal structures from twelve organisms, with or without cofactors and/or substrates/inhibitors, are deposited in the Protein Data Bank (PDB) (Table S2, Supplementary Materials). The crystal structures of DXR co-crystallized with inhibitors in the catalytic site provided key information on both the active site architecture and the binding mode of NADPH, DXP, and inhibitors.

#### Active Site 

Since DXR from *P. falciparum* is an attractive target for inhibitor design, we have used it for our analysis of both the active site and binding mode of fosmidomycin (1, Figure 6B). However, due to the N-terminal insertion of ca. 70 amino acids in *Pf*DXR (Figure 6A), the residue number of *Pf*DXR significantly differs from other DXRs. So, in the following description, *Ec*DXR numbering is shown in parentheses.

**Figure 7:**
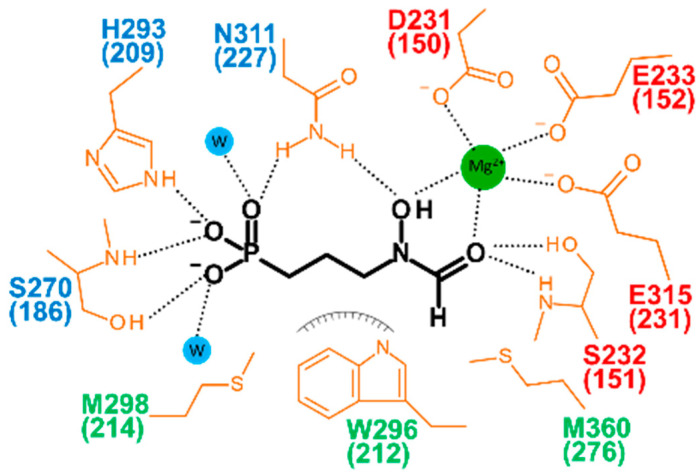
The binding mode of fosmidomycin in the active site of *Pf*DXR. Residues involved in the inhibitor binding are colored as in Figure 6A. The number in the parentheses indicates the residue number for the equivalent residue of *Ec*DXR.

The substrate-binding cavity of DXR is highly conserved in all organisms and consists mainly of three regions: a positively charged phosphate/phosphonate binding pocket, a hydrophobic region around the linker backbone, and a metal binding pocket [31,32]. Note that the residues involved in inhibitor binding described below are conserved among DXRs (Figure 6A). The substrate and substrate-analogous inhibitors bind to the cleft of the catalytic domain and induce a conformational change that tether the N-terminal and the catalytic domains in the closed conformation. Concomitantly with the movement of the catalytic domain, the C-terminal domain also shows a closed conformation. In addition, the flexible loop (residues 291–299 in *Pf*DXR, colored orange in Figure 6) in the catalytic domain adopts a conformation that allows it to function as a lid over the active site. The highly conserved residues Trp296 (212), Met298 (214), and Met360 (276) form a barrier between the active site and the solvent. The indole ring of Trp296 (212) provides the key hydrophobic interaction with the alkyl chain of the substrate and the backbone of fosmidomycin, which lies parallel within a distance of 4 Å. The acidic residues Asp231 (150), Glu233 (152), and Glu315 (231) are conserved at the active site and coordinate the divalent metal cation essential for enzyme activity. Met298 (214), Met360 (276), and the nicotinamide ring of NADPH also contribute to the formation of the hydrophobic binding pocket. The phosphonic acid moiety of fosmidomycin is bound similarly to the phosphate group of DXP in *Ec*DXR, forming hydrogen bonds with Ser270 (186) and Asn311 (227). The phosphonate group also forms a hydrogen bond with His293 (209). The hydroxamic acid moiety of fosmidomycin coordinates the divalent metal cation that is bound by the side chains of Asp231 (150), Glu233 (152), and Glu315 (231). The hydroxamate group also interacts with Ser232 (151) and Asn311 (227) (Figure 7). 

**Figure 8:**
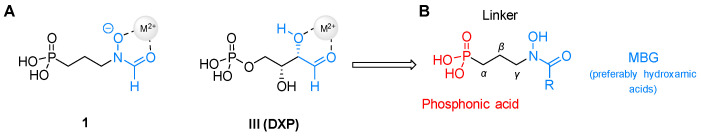
(**A**) Binding of **1** and natural substrate DXP (**III**) to a metal ion, represented by a grey sphere. (**B**) Simplified pharmacophore model of fosmidomycin-based DXR inhibitors MGB: Metal-binding group.

The hydroxamic acid moiety of fosmidomycin (**1**) mimics the hydroxyl ketone structure and the phosphonic acid the monoalkyl phosphonate structure of DXP (**XII**, Figure 8A). Therefore, the substrate analog fosmidomycin binds in the active site with a comparable binding mode. Fosmidomycin acts as a slow, tight-binding competitive inhibitor with the substrate while acting uncompetitively towards the cofactor NADPH [84]. Based on the structure and interaction of fosmidomycin and its analogs with the binding site of DXR, this class of inhibitors can be described by a pharmacophore model presented in Figure 8B.

## 4. Structural Modifications of Fosmidomycin and FR900098

Based on the pharmacophore model defined in the previous section (Figure 8B), modifications of fosmidomycin and FR900098 will be discussed. These structural changes to both lead structures were introduced to overcome their poor pharmacokinetic properties and to especially improve the permeability. To assess structure–activity relationships (SAR), a wide array of structural modifications will be presented as well as their impact on the anti-infective activity. Docking studies and co-crystal structures are included to further illustrate this SAR.

### 4.1. Modifications of the Retro-Hydroxamate Moiety

Chemically, fosmidomycin is often described as a retro-hydroxamate. More specifically, with respect to the hydroxamate moiety, fosmidomycin is an *N*-substituted formohydroxamic acid. Regarding fosmidomycin analogs, the term reverse fosmidomycin derivative is commonly used for analogs, where the carbonyl group of the reversed hydroxamic acid is attached to the propyl linker and not to the nitrogen. The hydroxamic acid (HA) functionality is a common bidentate metal binding group (MBG) capable of chelating metal cations such as Zn^2+^, Fe^2+^, Fe^3+^, Mg^2+^ and Mn^2+^ in the active sites of metalloenzymes [96]. The chelation of the catalytically essential metal cation (Mg^2+^ or Mn^2+^) in the active site of DXR by the retro-hydroxamate group of fosmidomycin is essential to its anti-infective effects. 

#### 4.1.1. Inversion of the Retro-Hydroxamate Moiety

**Figure 9:**
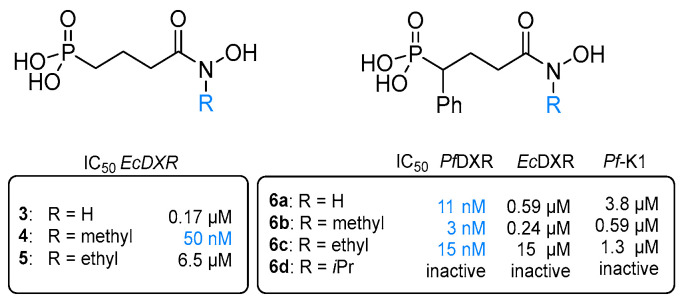
Structures, antibacterial, and antiplasmodial activities of the reverse fosmidomycin analogs **3**–**6**.

The concept of reversing the orientation of the hydroxamate moiety was pioneered by the Rohmer group which synthesized compounds **3** and **4** (Figure 9) as analogs of fosmidomycin and FR900098. Both reverse analogs exhibited inhibitory activity comparable to that of fosmidomycin against *Ec*DXR with IC_50_ values of 0.17 (**3**) and 0.05 µM (**4**), respectively [97]. One year later, Woo et al. showed that compound **3** is a slower *Synechocystis* DXR binder in comparison to fosmidomycin [98]. In 2010, Zinglé et al. demonstrated that the superior *Ec*DXR inhibition of **4** was attributed to the hydrophobic interaction between the *N*-methyl group and the indole of Trp212 of *Ec*DXR [99]. Homolog **5** with an ethyl residue showed two orders of magnitude decrease in activity compared to **4 [99]**.

In parallel, reverse fosmidomycin analogs with a phenyl substituent in the *α*-position of the propyl linker were reported by Kurz and co-workers (**6a**–**d**, Figure 9). *α*-Phenyl analog (**6a**) served as a lead compound for the reverse inhibitor type. Furthermore, the compounds were decorated with small alkyl substituents (Me, Et, *i*Pr) at the hydroxamic acid nitrogen. The *N*-methylated carba analog **6b** was the most active inhibitor in the first reverse series, outperforming fosmidomycin and FR900098 in *Ec*DXR and *Pf*DXR inhibition with IC_50_ values of 0.24 μM and 3 nM, respectively. Enzyme inhibition data demonstrated that the strength of *Ec*DXR and *Pf*DXR inhibition decreased as the size of the substituent on the hydroxamic acid nitrogen increased. While the *N*-methyl-substituted DXR inhibitor **6b** is a potent *Pf* growth inhibitor, the *N*-ethyl substituted derivative **6c** already showed a 5-fold reduction in cellular antiplasmodial activity against *Pf*-K1. The bulkier *N*-isopropyl group of compound **6d** led to a loss of inhibitory activity against the *Pf*DXR and *Ec*DXR enzymes and *Pf*-K1 [100,101].

#### 4.1.2. Alteration of the Acyl Moiety and Replacement of the Hydroxamic Acid Moiety

**Figure 10:**
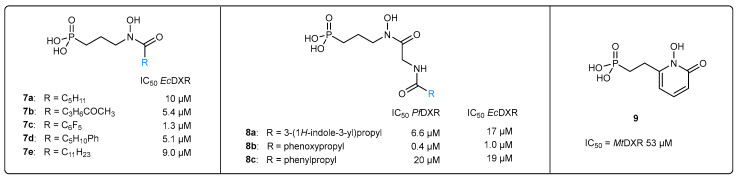
Structures and biological activities of compounds **7***–***9** with modified MBGs.

In order to create beneficial hydrophobic interactions in the hydrophobic sub-pockets of *Ec* and *Pf*DXR, Giessmann et al. [102] and Ortmann et al. [103] replaced the formyl group of fosmidomycin with aliphatic and aromatic acyl residues (**7, 8**, Figure 10). Within these series, the pentafluoro benzoyl derivative (**7c**) and the 4-phenoxybutanamide analog (**8b**) were the most active representatives with IC_50_ values of 1.3 (**7c**) and 1.0 µM (**8b**) against *E*cDXR [102,103]. Flexible docking studies suggested that the acyl residues of compounds **7a**–**e** prevented the formation of the preferred geometry of the hydroxamate-metal complex [103].

Andaloussi et al. developed the *N*-hydroxypyridone **9** (Figure 10) as an MBG [104], which only showed very weak *Mt*DXR enzyme inhibition with an IC_50_ value of 53 µM and no in vitro growth inhibition of *Mt* [104].

**Figure 11:**
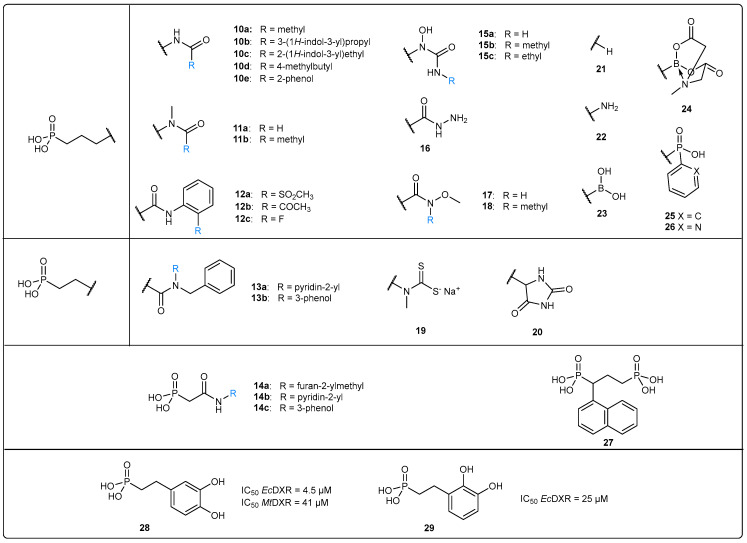
Structures and biological activities of analogs **10***–***29** with alternative chelating functionalities.

To confirm the importance of the hydroxamic acid functionality, the hydroxamic acid MBG was replaced with various amide moieties (**10a**–**e**, Figure 11) [102,105]. The IC_50_ values of amides **10a**–**e** against *Ec*DXR was > 30 µM. This was also demonstrated for fosmidomycin and FR900098 by Woo et al. who replaced the *N*-hydroxyl group of the retro-hydroxamate moiety with a methyl group in compounds **11a**, **b** (Figure 11) resulting in a complete loss of inhibitory activity against *Synechocystis* DXR [98]. Chofor et al. reported a series of *ortho*-substituted arylamide derivatives (**12a**–**c**, Figure 11) [16]. These *ortho*-substituents were expected to contribute to the chelation of the active site metal cation. However, none of the synthesized derivatives **12a**–**c** inhibited *Ec*DXR and *Mt*DXR at a concentration of 100 µM nor the growth of *Pf*-K1 parasites in human erythrocytes. According to Chofor et al., the low flexibility of the amide bond might be responsible for the lack of metal-binding and, therefore, inhibitory activity [16].

Kaye and colleagues studied the replacement of the hydroxamate MBG with a variety of *N*-arylalkyl substituted amides (**13a, b**, Figure 11), in which the benzyl group was intended to occupy the hydrophobic sub-pocket of the substrate binding site. However, the synthesized analogs **13a, b** were completely inactive against the *Pf*DXR enzyme [106]. Secondly, they introduced aryl and heteroaryl carboxamide groups (**14a**–**c**, Figure 11) in addition to shortening the propyl linker, but these modifications also led to inactive analogs [107,108].

Further work on replacing the hydroxamate MBG with different nitrogen-containing metal chelating moieties such as hydroxyureas (**15a**–**c**), hydrazide (**16**), *O*-methylated hydroxamate (**17**, **18**), dithiocarbamate (**19**) or hydantoin (**20**) functionalities (Figure 11) was performed [109]. For the hydroxyureas (**15a**–**c**) results regarding antiplasmodial or antibacterial activity have not been published [109]. Furthermore, the potential metal chelators **16**–**20** did not display any inhibitory activity towards *Ec*DXR. The authors suggested that the protonation of the hydrazide group of **16** under the assay’s conditions (pH = 7.5) could explain the loss of its chelation capability. In the case of **19**, the authors reported decomposition of the dithiocarbamate moiety under the conditions of the enzyme assay [110]. The negligible *Mt*DXR inhibition of **20** is not surprising given that the hydantoin moiety of derivative **20** is not an established MBG [104]. Mercklé et al. showed that, as expected, the removal of the hydroxamic acid MBG as in the propyl phosphonic acid **21** and the aminopropyl phosphonic acid **22** (Figure 11) resulted in a complete loss of activity towards *Ec*DXR [105]. Mancini reported a chemically interesting derivative with a boronic acid unit as a potential MBG (**23**, Figure 11), though it was inactive in the *Ec*DXR enzyme assay. Prodrug **24** (Figure 11) to boronic acid **23** showed negligible activity towards *E. coli* [111]. Additionally, phosphinic acids with an aryl (**25**) or heteroaryl residues (**26**) were synthesized, but not evaluated against DXR (Figure 11) [112]. Furthermore, the bisphosphonic acid (**27**) was also inactive against DXR of *Ec*, *Pf* and *Mt* (Figure 11) [93]. The two catechol derivatives with a 3,4-catechol (**28**) and 2,3-catechol moiety (**29**) stood in contrast to the previously mentioned inactive derivatives (Figure 11). Compound **28** was at least weakly active against *Mt*DXR (IC_50_ = 41 µM) but no in vitro growth inhibition of *Mt* was observed. Interestingly, when tested against *Ec*DXR, the 2,3-catechol derivative **29** (IC_50_ = 25 μM) was weaker than the 3,4-catechol analog **28** (IC_50_ = 4.5 μM). These results confirmed the importance of the position of the two catechol hydroxyl groups for sufficient metal coordination [110,113].

So far, all attempts to replace the hydroxamate group with alternative chelating groups greatly reduced or resulted in a complete loss of inhibitory activity. The above-summarized results illustrate the predominant role of the hydroxamic acid MBG in DXR inhibitors.

#### 4.1.3. Development of Bisubstrate Inhibitors

Since the adenosine-binding pocket of the cofactor NADPH returned a good score on a druggability test conducted by Hirsch and coworkers [114], a new bisubstrate inhibitor approach has been explored, aimed at simultaneously targeting the substrate and cofactor binding sites of DXR.

**Figure 12:**
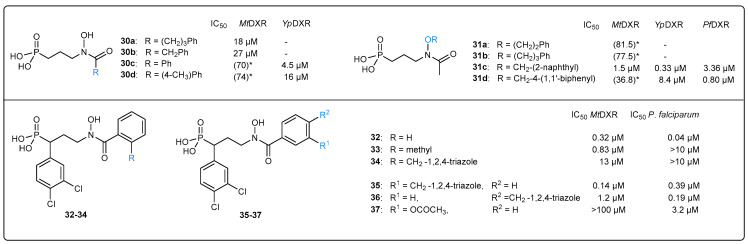
Biological activities of the potential bisubstrate inhibitors **30***–***37**. Values marked with * indicate the percentage of inhibition at 100 μM.

Guided by an *Mt*DXR fosmidomycin co-crystal structure, the Dowd group was the first to develop a series of fosmidomycin analogs aimed to occupy both binding pockets. Two series of compounds, fosmidomycin-like hydroxamic acids with large acyl residues (**30a**–**d**, Figure 12) and arylalkoxyamides (**31a**–**d**, termed *O*-linked bisubstrate inhibitors, Figure 12), were synthesized as potential bisubstrate inhibitors. While none of the derivatives was more active than fosmidomycin against *Mt*DXR, compounds **30a** and **30b** showed at least weak IC_50_ values of 18 and 27 µM. Docking experiments suggested that compound **30a** could interact with *Mt*DXR via an alternative non-bisubstrate mode of binding. So far, the Dowd group concluded that the hydroxamic acids **30a, b** are more potent *Mt*DXR inhibitors than the arylalkoxyamide derivatives (**31a, b**) [41,115]. Later on, compound **30a** was tested for its ability to inhibit the *Pf*DXR enzyme, but the compound showed only moderate activity with an IC_50_ of 1.34 µM [115,116].

To improve the antibacterial activity and confirm that both hydroxamic acid and arylalkyloxyamide analogs can act as DXR bisubstrate inhibitors a larger series of hydroxamic acids (**30c, d**, Figure 12) and arylalkyloxyamides (**31c, d**, Figure 12) was developed by San Jose et al. [41]. When tested against *Mt*DXR, the most active compound was the arylalkyloxyamide **31c** with an IC_50_ value of 1.5 μM. However, **31c** and **31d** required concentrations of ≥200 μg/mL to be effective against *Mt*, while compound **31d** inhibited the growth of *Mt* at 25–50 μg/mL [41]. With an IC_50_ value of 0.33 μM against *Yp*DXR, compound **31c** was the most potent analog. Therefore, the authors suggested that a free hydroxamic acid functionality to strongly chelate the metal cation is not necessary for *Yp*DXR inhibition in the case of these potential bisubstrate inhibitors [41]. To assess whether **30c** and **31c** are bisubstrate inhibitors, Lineweaver-Burk analysis of **30c** and **31c**, tested against *Mt*DXR and *Yp*DXR, respectively, indicated that both compounds competitively inhibit NADPH and DXP. In 2021 Girma et al. tested alkoxyamides **31c, d** for their activity against *Pf*DXR. **31d** showed superior activity to **31c** with an IC_50_ of 0.80 to 3.36 μM, respectively. To determine the mechanism of inhibition of **31d**, Lineweaver-Burk analysis against *Pf*DXR enzyme was also performed. **31d** showed the lowest inhibition constant (K_i_) with respect to both the substrate DXP and the cofactor NADPH. This finding demonstrated that **31d** is a bisubstrate inhibitor of *Pf*DXR [116].

The retro-hydroxamate **32** (Figure 12) showed submicromolar inhibitory activity towards *Mt*DXR (IC_50_ = 0.32 μM) in addition to potent in vitro growth inhibition in a *Pf* parasite assay (IC_50_ = 0.04 μM). A co-crystal structure of **32** in complex with *Mt*DXR in the presence of NADPH (PDB 3ZHY) showed that the terminal phenyl ring binds close to the NADPH binding site at a distance of 3.5–3.7 Å of the NADPH nicotinamide ring. In this position, the terminal phenyl ring can also interact with Met267. Inspired by the crystal structure, additional substituents were introduced at different positions on the phenyl ring, aimed at reaching the cofactor-binding pocket (**32**–**34**, Figure 12). The presence of methyl (**32**) or 1,2,4-triazole **(33**) substituents in the *ortho*-position of the phenyl moiety did not improve the inhibition. Isomers **35** and **36** (Figure 12) featuring the 1,2,4-triazole substituent in the *meta*- and *para*-position displayed enhanced inhibitory activity against *Mt*DXR compared to **34**, with IC_50_ values of 0.14 μM (**35**) and 1.2 μM (**36**).

In contrast, the introduction of the phenol ester substituent in compound **37** (Figure 12) resulted in a complete loss of activity towards *Mt*DXR. The strong in vitro growth inhibition of compounds **35** and **36** in a *Pf* growth assay and their potent *Mt*DXR inhibition suggested that the terminal triazole moiety might be involved in specific interactions with the DXR binding site [117]. Current efforts to develop DXR bisubstrate inhibitors provided novel compounds with heterogenous biological activities, with some inhibitors (**32**, **35**) showing very promising *Mt*DXR inhibition and antiplasmodial in vitro activity. Analog **37** showed that larger residues within the NADPH binding site are tolerated. This provides an interesting starting point for the development of further inhibitors of this class. To further elucidate the binding modes of potential bisubstrate inhibitors, co-crystallization with an occupied NADPH binding site is required.

### 4.2. Modifications of the Propyl Linker 

Earlier studies already highlighted the importance of the phosphonic acid and hydroxamate groups and a well-defined linker length between both pharmacophores. In contrast, the linker modifications provided a wide spectrum of options for further improvements of anti-infective activity against various microorganisms. The synthesized derivatives are structurally diverse. These modifications encompass alterations of the linker length (**38**–**41**), insertion of a double bond (**42**–**46**) or hetero atoms (**47**–**54**), restriction of the linker flexibility (**55**–**60**) and substitution of the linker in the *α*-, *β*- and *γ*-position. 

#### 4.2.1. Linker Length Variation 

**Figure 13:**
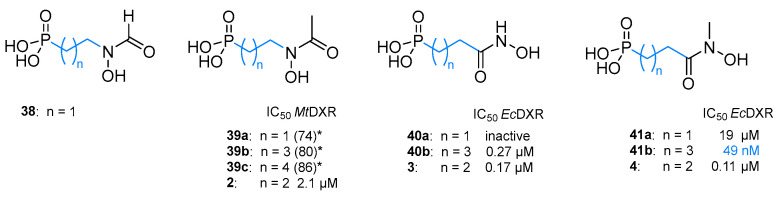
Biological activities of analogs **38***–***41.** Values marked with * indicate the percentage of inhibition at 100 μM.

In an initial effort to determine the ideal linker length, the Fujisawa Pharmaceutical Co., Ltd. was the first to explore modifications of the carbon backbone. However, the shortened ethylene analogs **38** did not show any antibacterial activity against, e.g., *P. aeruginosa* (Figure 13) [118]. The Dowd group synthesized a series of FR900098 analogs with two to five methylene units separating the MBG and phosphonic acid moiety (**39a**–**c**, Figure 13) [119]. The results showed that compounds with chain lengths of two, four or five methylene groups weakly inhibited *Mt*DXR at 100 µM (74-86%) [119]. Later, Zinglé et al. prepared reverse fosmidomycin (**3**) and reverse FR900098 (**4**) homologs bearing a shortened ethylene (**40a, 41a**, Figure 13) or extended butylene linker (**40b, 41b**, Figure 13). The derivatives with an ethylene linker were weakly active (**41a**) or lacked activity (**40a**) against *Ec*DXR, while the reduction was less drastic for the butylene homologs (**40b, 41b**), with IC_50_ values of 0.27 and 0.11 μM, respectively [99].

#### 4.2.2. α,β-Unsaturated Propenyl Linker 

FR32863 (**IV**), a natural antibiotic isolated from *Streptomyces lavendulae* in 1980, is the dehydro-congener of fosmidomycin (**1**). FR32863 and its acetylated analog **42** showed activity similar to **1** and **2** against a panel of Gram-negative bacteria including *P. aeruginosa* [21,118]. Biological evaluation showed that FR32863 is an excellent inhibitor of *Pf*DXR (IC_50_ = 9 nM) that also potently inhibits the growth of *Pf*3D7 with an IC_50_ of 19 nM. Compound **42** was further active against *Mt*DXR with an IC_50_ of 1.1 µM. Based on these results, Devreux synthesized *Z*- (**43a**–**e**, **44d**) and *E*-configured (**44a**–**c**, Figure 13) *α,β*-unsaturated DXR inhibitors with additional substituents in the *α*-position and tested them against *Ec*DXR [119]. The *Z*-configured *α*-bromo derivative **44d** was the most active compound, displaying a submicromolar IC_50_ value of 0.45 µM. Furthermore, the *E-*configured.

**Figure 14:**
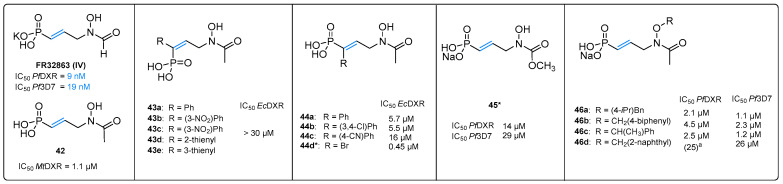
Biological activity of the fosmidomycin and FR900098 derivatives (**42**–**46**) with an *α,β*-unsaturated linker. * Ammonium salts were prepared. ^a^ Values in parentheses are the percent remaining enzyme activity at 100 μM.

Analogs (**44a**–**c**) were active in the micromolar range (IC_50_ = 5.5–16 µM), while the *Z*-configured derivatives **43a**–**e** were inactive. This suggested that the relative *trans*-conformation of the phosphonic acid and hydroxamate moiety towards each other is essential for activity. However, the combination of an *α*-substituent and an *α, β-*unsaturated linker constrained the rotational freedom, thus leading to reduced activity against *Ec*DXR [120]. The Dowd group also designed and synthesized a series of FR32863 (**IV**) analogs **45** and **46a**–**d** (Figure 14). Although, none of the derivatives exceeded the activity of FR32863 against *Pf*DXR, compounds **45** and **46a**–**c** showed moderate inhibitory activity in vitro with IC_50_ of 2.1–14 µM [121].

#### 4.2.3. Oxa Analogs

**Figure 15:**
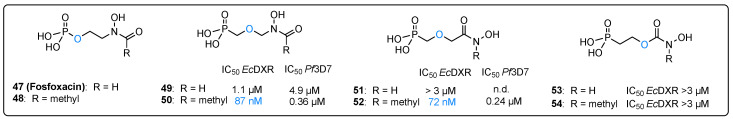
Structure and biological activity of the oxa analogs **47***–***54**.

Fosfoxacin (**47**, Figure 15), the naturally occurring phosphate congener and *α*-oxa analog of fosmidomycin as well as fosfoxacin’s acetyl analog (**48**), are more potent inhibitors of *Synechocystis* DXR compared to fosmidomycin [122]. These results encouraged Haemers et al. to synthesize a series of *β-* and *γ-*oxa-isosteres (**49**–**54**, Figure 15) as the electronegative oxygen might increase the acidity of the phosphonic acid and hydroxamic acid moiety. While compounds **49** and **50** are *β-*oxa-isosteres of fosmidomycin and FR900098, respectively, analogs **51**–**54** are reverse derivatives. The results showed that the *β*-oxa analogs **49**–**52** are more active *Ec*DXR inhibitors than the *γ*-counterparts (hydroxycarbamates) **53** and **54**. The *β*-oxa isosteres **50** and **52** were almost as potent as FR900098 displaying IC_50_ values of 87 nM (**50**) and 72 nM (**52**) against *Ec*DXR and submicromolar activity against strain *Pf*3D7 [123].

#### 4.2.4. Conformationally Restricted Analogs

**Figure 16:**
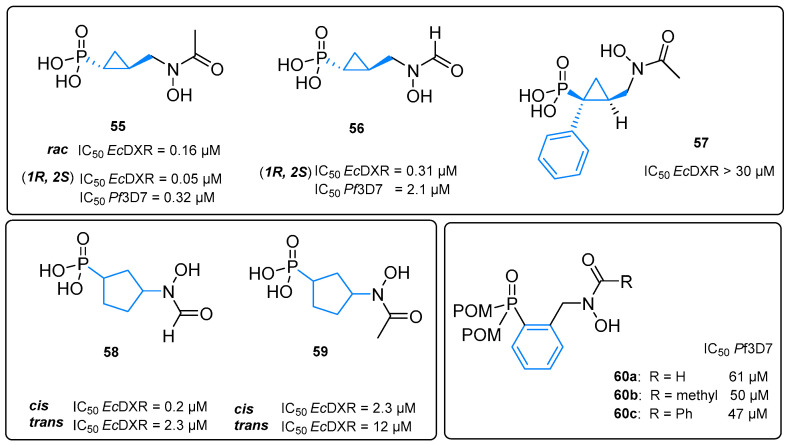
Biological activity of conformationally restricted analogs **55***–***60**.

In 2006, the Van Calenbergh group incorporated a cyclopropyl (**55**–**57**, Figure 16) [124] and cyclopentyl (**58**–**59**, Figure 16) [125] ring into the linker to restrict rotational freedom. The racemic *trans*-cyclopropyl *N*-acetyl analog **55** resulted in submicromolar inhibition of *Ec*DXR with an IC_50_ of 0.16 µM, while the enantiomerically pure (1*R*,2*S*)-**55** showed enhanced activity (IC_50_ = 50 nM) that was comparable to fosmidomycin. Additionally, (1*R*,2*S*)-**55** was similarly active compared to fosmidomycin (IC_50_ = 0.32 µM) in an in vitro *Pf*3D7 assay. The replacement of the acetyl moiety of (1*R*,2*S*)-**55** by a formyl moiety (**56**) reduced the activity against *Ec*DXR and *Pf*3D7 by approximately 6-fold. 

The racemic mixture of an *α*-phenyl substituted *cis*-cyclopropyl derivative (**57**) was inactive towards *Ec*DXR. Unfortunately, it is not clear whether the loss of activity was caused by the bulky *α*-phenyl moiety or by the *cis*-conformation of the phosphonic acid and hydroxamate moieties [124]. Haemers et al. gave some insights regarding the preferred configuration of restricted analogs as they synthesized the *cis*- and *trans*-cyclopentyl derivatives of fosmidomycin (**1**) and FR900098 (**2**). The *cis* isomers of **58** and **59** (IC_50_ values of 0.20 and 2.3 µM, respectively) were more active than their *trans*-isomers (IC_50_ values of 2.3 and 12 µM, respectively).

One year later, the synthesis and antiplasmodial activity of three conformationally restrained aromatic analogs **60a**–**c** (Figure 16) were reported by Walter and colleagues. The analogs **60a**–**c** were tested as bis(pivaloyloxymethyl) (POM) ester prodrugs and the POM prodrugs of **1** and **2** were prepared for direct comparison. While the POM-prodrugs of **1** and **2** were moderately active against *Pf*3D7 (IC_50_ = 0.4–2.1 µM), the activity of the corresponding rigidized analogs (**60a**–**c**) was very weak [126].

### 4.3. α-, β- and γ-Substituted Fosmidomycin Analogs

#### 4.3.1. α-Phenyl and α-Biaryl-Substituted Analogs

**Figure 17:**
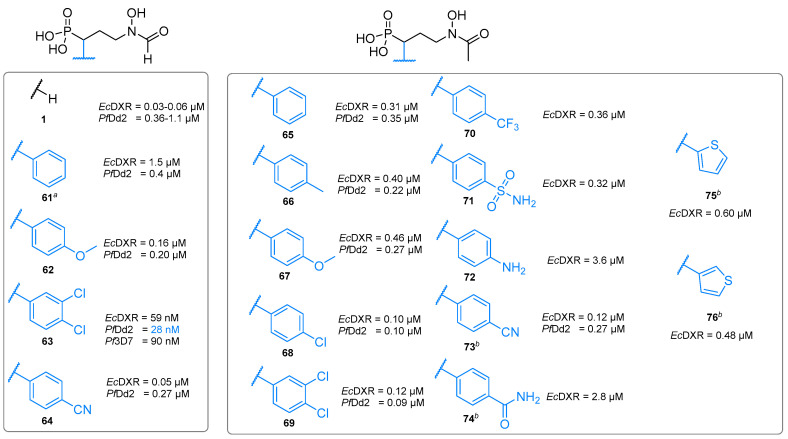
Antibacterial and antiplasmodial activities of α-phenyl derivatives (**61***–***76**). ^a^ Diethanolammonium salt was prepared. ^b^ Ammonium salts were prepared.

To date, most attempts to improve the anti-infective properties of fosmidomycin are related to the substitution and modification of the *α*-position of the propyl linker (Figure 17) [45,125,127,128,129,130,131]. The first chain-substituted derivatives were decorated with an *α-*phenyl-substituent and were synthesized and patented in 2005 by Kurz et al. [132]. The diethanolammonium salt of *α*-phenyl-fosmidomycin (**61**, Figure 17) was the first inhibitor to exceed the antiplasmodial activity of FR900098 (**61** IC_50_ = 0.4 µM and FR900098 IC_50_ = 0.8 µM) against *Pf*Dd2.

In 2006 and 2007, Van Calenbergh and coworkers presented a comprehensive series of *α*-phenyl substituted analogs with electron-rich and electron-deficient substituents at the phenyl moiety (**62**–**74**, Figure 17) [130]. The authors aimed to investigate the influence of lipophilicity and electronic properties with respect to their inhibitory activity against *Ec*DXR and the *Pf*Dd2 strain [45]. Whereas several *α*-phenyl-substituted compounds of this series showed slightly lower inhibitory activity than lead structures fosmidomycin (**1**) and FR900098 (**2**) against *Ec*DXR, the inhibition of *Pf* growth (Dd2 and 3D7 strains) was consistently superior. The authors suggested the 3,4-dichlorophenyl unit of the derivatives **63** and **69** increased the lipophilicity and facilitated entry into *P. falciparum* cells and/or enabled more selective interactions with *Pf*DXR. The *α*-3,4-dichlorophenyl substituted analog **63** was a milestone in lead optimization, as it was the first inhibitor which exceeded the potency of **2**, with an IC_50_ value of 59 nM against *Ec*DXR. In addition, **63** exhibited potent in vitro activity with IC_50_ values of 28 and 90 nM against *Pf*Dd2 and *Pf*3D7 strains, respectively. Unfortunately, *Pf*DXR inhibition was not reported [45].

In accordance with previous studies [45], the potency of the most active *N*-acetyl-(4-cyano)phenyl analog **73** was exceeded by its *N*-formyl analog **64**. Furthermore, both compounds exceeded **1** and **2** in an antiplasmodial growth assay (IC_50_ = 0.27 µM) using intraerythrocytic stages of the *Pf*D2d strain [130]. A 2-thienyl and 3-thienyl analog (**75**–**76**, Figure 17) exhibited submicromolar activity (IC_50_ = 0.48–0.60 μM) as well, which confirmed that thiophene can be used in this case as a phenyl bioisoster [130].

Achieving whole-cell activity for fosmidomycin-like DXR inhibitors against *M. tuberculosis* is particularly challenging as *Mt* lacks the GlpT-type transporters, responsible for inhibitor uptake in other bacteria.

**Figure 18:**
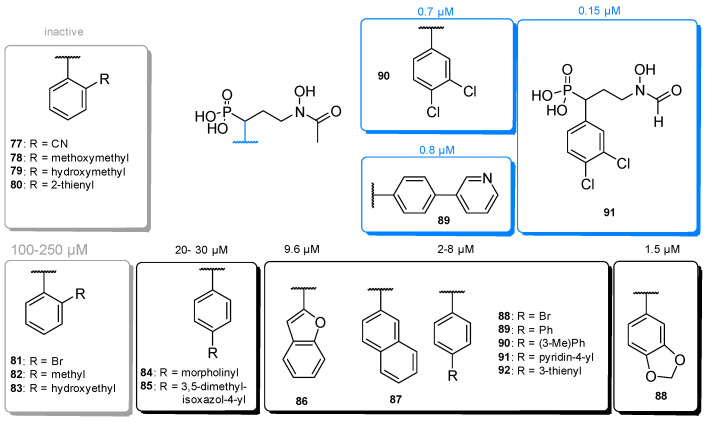
Structure–activity relationship of α-substituted FR900098 analogs **77**–**90**, the *N*-formyl analog **91** and their activity against *Mt*DXR.

**Figure 19:**
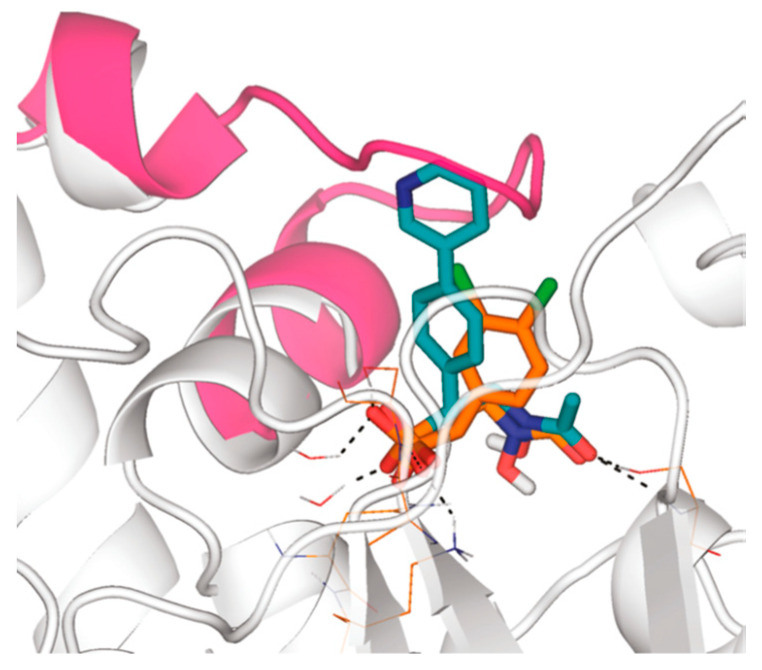
Compound **89** (turquoise carbon atoms) docked in the X-ray structure of *Mt*DXR in complex with 3 orange carbon atoms (PDB code 2Y1G) [128]. The Gly198-Met208 flap (colored in pink) from the 2JVC structure representing *Mt*DXR bound to fosmidomycin (**1**). Reprinted with permission from *J. Org. Chem. 2011, 76, 21, 8986–8998* [129]. Copyright 2022 American Chemical Society.

In 2011, FR900098 (**2**) was first tested against *Mt*DXR by the Karlén group [128]. Although significant *Mt*DXR inhibition was observed, no antimycobacterial activity was detected. In response, the Karlén group developed more lipophilic inhibitors based on the work of Van Calenbergh and Kurz [120,130,131,133]. Both research groups demonstrated that *α-*phenyl substituents increased the activity of *Pf* and *Ec*DXR inhibitors. Employing the same strategy, the Karlén group synthesized several *α-*phenyl derivatives with different substituents in the *ortho*-position of the phenyl moiety, *α-*biaryl derivatives, and inhibitors with bicyclic ring systems in the *α*-position (**77**–**91**, Figure 18) [128,129]. The *ortho*-substitution of the *α-*phenyl moiety completely reduced the activities of the inhibitors against *Mt*DXR. Docking experiments proposed possible clashes between the *ortho*-substituents and the enzyme, which could explain the loss of activity. Furthermore, none of the derivatives (**77**–**91**, Figure 18) inhibited the growth of *Mt* H37Rv. The inhibitors with bulky moieties in the *α*-position (**84**–**88**) inhibited *Mt*DXR with IC_50_ values between 1.5 and 27 µM. The *α-*4-(pyridine-3-yl)phenyl analog (**89**) exhibited the best activity against *Mt*DXR (IC_50_ = 0.8 µM), which is comparable to the *α*-3,4-dichlorophenyl derivate (**90**, IC_50_ = 0.7 µM). No correlation between calculated logP and IC_50_ values of this compound series (**77**–**91**) was found. Docking experiments of **89** with *Mt*DXR suggested that the biaryl moiety of **89** interacts with the flexible loop formed by Gly198-Met208 (Figure 19). This possible interaction provided the basis for further inhibitor optimizations. 

#### 4.3.2. α-Halogenated Phosphonic Acid Derivatives

**Figure 20:**
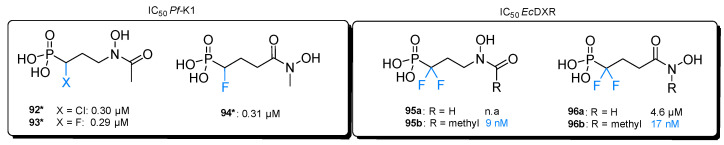
Biological activities of α-halogenated phosphonic acid derivatives (**92***–***96**). * Ammonium salts were prepared.

Verbrugghen et al. [127] tried to mimic the acidity of the phosphate group of fosfoxacin (**47**, Section 4.2.3) with *α*-chloro (**92**) and *α*-fluoro (**93**) phosphonic acid moieties (Figure 20). Additionally, the group conducted P^31^-NMR-titrations of FR900098 and its *α*-chloro and fluoro analogs (**92, 93**). This experiment revealed that the decreased pKa_2_ value of the phosphonic acid moiety of both analogs (pKa_2_ ~ 6 vs. pKa_2_ ~ 7.35 for **2**) is isoacidic to a monoalkyl phosphate group. The corresponding SAR data led to the conclusion that DXR inhibitors with a dianionic phosphonate group are more potent inhibitors than the corresponding monoprotonated phosphonate anions (further explanations are discussed in Section 5) [98,104,127,134].

Furthermore, the Van Calenbergh group synthesized the *α*-fluoro analog (**94**) of the reverse FR900098 [127]. The three racemic compounds (**92**–**94**) were screened for their activity against asexual blood stages of *Pf* and found to inhibit their growth with IC_50_ values in the micromolar range (IC_50_ = 0.29–0.31 µM), surpassing the activity of FR900098 (IC_50_ = 0.42 µM). Both fluorinated analogs (**93**–**94**) were further evaluated in the *Plasmodium berghei* (GFP ANKA strain) mouse model by intraperitoneal (i.p.) application of high doses (50 mg/kg) for 5 consecutive days. Chloroquine (CQ) eradicated parasitemia after 4 days post-infection, while FR900098 only led to 93% suppression of parasitemia. Compared to the reference substances CQ and FR9800098, the in vivo activity of **93** (88%) and **94** (85%) was slightly weaker on day 4. In summary, **93** and **94** exhibited significant in vivo antimalarial activity at day 4 after i.p. application, but none of the monohalogenated DXR inhibitors (**93**–**94**) demonstrated curative antimalarial activity [127]. This was an important outcome and provided a starting point to investigate inhibitors with further substitutions at the *α*-methylene group.

Recently, Dreneau et al. extended the mono *α*-halogenation into difluorination synthesizing the *α,α*-difluorophosphonic acid derivatives of fosmidomycin and FR9000098 (**95a, b**, Figure 20) as well as their reverse analogs (**96a, b**, Figure 20). The difluorinated analogs were tested for their inhibitory activity against *Ec*DXR and a fosmidomycin-resistant *E. coli* (FosR) strain [135]. Against *Ec*DXR, the *N*-acylated and *N*-methylated derivatives (**95b**, **96b**) showed excellent IC_50_ values of 9 and 17 nM, respectively. Therefore, the potency of **95b** and **96b** is in the same range as fosmidomycin and FR900098. In contrast, the non-methylated difluoromethylene compound **96a** was significantly less efficient than its *N*-methylated congener **96a** (IC_50_ = 4.6 µM) against *Ec*DXR. The same observation was made in the *E. coli* growth inhibition assay paper determined with the disc diffusion method, where **96a** was inactive, while **95b** and **96b** effectively inhibited bacterial growth. Moreover, no spontaneous resistance to these compounds occurred in *E. coli* as was observed for fosmidomycin [136]. This increase in activity was attributed to the formation of phosphonate dianions under test conditions. The isoacidic nature and the isosteric geometry of the fluorinated phosphonic moiety, together with improved electrostatic and van der Waals interactions, are possible explanations for the pronounced activity. None of the derivatives could prevent the growth of the fosmidomycin-resistant *E. coli* strain FosR, in which the GlpT transporter is dysfunctional and did not facilitate the uptake of these inhibitors. This suggested that uptake of inhibitors **95a, b** and **96a, b** relied on an active transport mechanism by intact and functioning GlpT transporters. In summary, the introduction of two fluorine atoms in the *α-*position of the linker improved *Ec*DXR inhibition significantly and enhanced the antimicrobial activity compared to phosphate analogs (**47**, **48**) or non-fluorinated lead structures in *E. coli* (**1**–**4**, Figure 9). 

#### 4.3.3. Structurally Diverse Substituents in the α-Position

**Figure 21:**
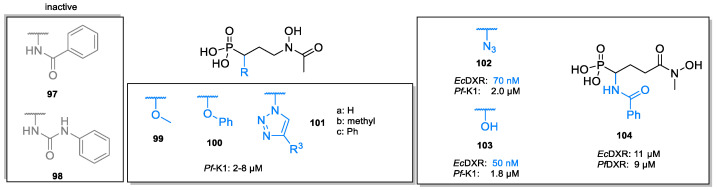
Biological data of structurally diverse *α*-substituted analogs **97**–**104**.

The promising results obtained with the *α*-phenyl substituted DXR inhibitors (Section 4.3.1), encouraged the Van Calenbergh group to extend the scope of *α*-substituents to benzamido (**97**), a phenylurea moiety (**98**), methoxy (**99**), phenoxy (**100**), substituted 1,2,3-triazolyl groups (**101a**–**c**), azido (**102**) and a hydroxyl group (**103**, all Figure 21) [137]. Of the structurally diverse inhibitors, only the *α*-azido derivative (**102**) and the *α*-hydroxy derivative (**103**) showed pronounced *Ec*DXR inhibition. The electron-rich *α*-triazole derivatives (**101a**–**c**) did not inhibit *Ec*DXR and only moderately suppressed the growth of *Pf*-K1. This behavior was postulated by the authors to be caused by the inability of the triazole ring to form π–π interactions with Trp211. Later, a reverse analog (**104**, Figure 21) was synthesized and showed weak inhibition of *Pf* and *Ec*DXR with IC_50_ values of 9–11 µM [138].

**Figure 22:**
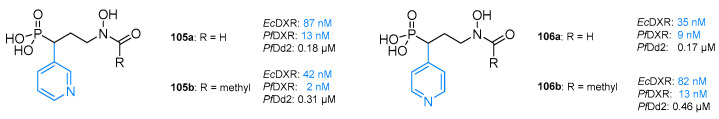
Rational design of α-pyridinyl DXR-inhibitors **105a, b** and **106a, b**. K_i_ values are given in µM (black) or nM (blue) against *Ec*DXR, *Pf*DXR and *Pf*Dd2.

The *α*-pyridinyl-substituted fosmidomycin analogs (**105a, b** and **106a, b**, Figure 22) were designed by Xue et al. [139] and assessed with respect to their inhibitory potential against *Ec*DXR, *Pf*DXR, *Pf*Dd2 and *Pf*3D7 (data of the latter not shown). The pyridine-containing derivatives **105a, b** and **106a, b** showed similar IC_50_ values to fosmidomycin when tested against *Ec*DXR (IC_50_ = 35–87 nM vs. IC_50_ (**1**) = 34 nM), while being 2-fold more active than fosmidomycin towards *Pf*DXR (IC_50_ = 2-13 nM vs. IC_50_ (**1**) = 21 nM). The antiplasmodial activity of the four compounds was stronger compared to fosmidomycin. Similar to fosmidomycin, the four pyridine derivatives (**105a, b** and **106a, b**) exhibited no cytotoxicity against human noncancerous fibroblast WI-38 cells (>300 µM) resulting in extraordinarily high selectivity indices of >1700.

**Figure 23:**
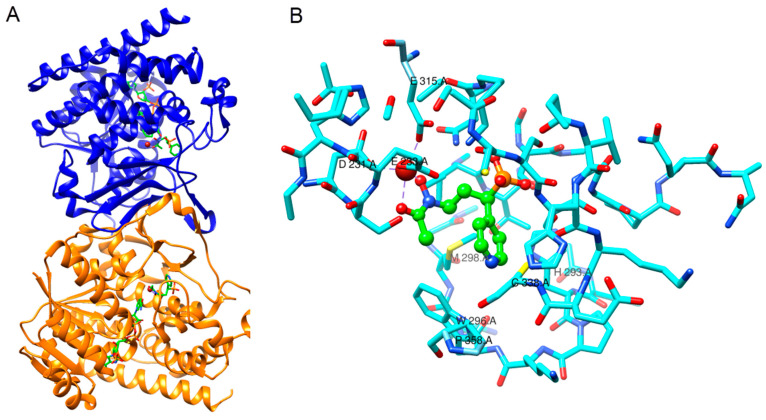
(**A**) overall structure of *Pf*DXR in complex with Mn^2+^ (brown sphere), **106b** (green) and NADPH. (**B**) Close-up view of the active site of *Pf*DXR:**106b**. Reproduced from Xue et al. (PDB: 4GAE [139].

To elucidate the interactions between **106b** and *Pf*DXR, the crystal structure in complex with NADPH and Mn^2+^ was solved and analyzed (Figure 23). In the crystal structure, the backbone of **106b** showed similar interactions to previously analyzed DXR inhibitors with *α*-phenyl substituents (e.g., **66**, Figure 17) [32,94,140,141]. In addition, the pyridine nitrogen atom formed a hydrogen bond with the thiol group of Cys338. This interaction is not possible for the unsubstituted phenyl analog **66** and is thus a conceivable explanation for the weaker activity of **66** compared to **106b**. 

#### 4.3.4. α-Substituted Reverse Carba Analogs

**Figure 24:**
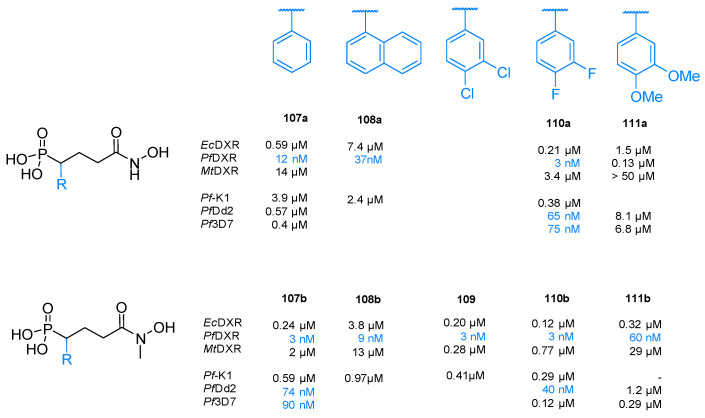
Biological data of *α*-substituted reverse fosmidomycin analogs (**105**–**109**).

Focusing on reverse fosmidomycin analogs, Kurz and coworkers broadly investigated the effects of the substitution at the *α*-position of the propyl linker [93,100,101]. The comprehensive biological data of *α*-substituted reverse analogs (**107**–**111**) are summarized in Figure 24. Several reverse carba analogs (**107b, 108a, 109** and **110a, b, 111b**) inhibited *Pf*DXR with IC_50_ values in the low nanomolar range and outperformed fosmidomycin. The IC_50_ values against *Ec*DXR are 1–3 orders of magnitude higher than the corresponding IC_50_ values for *Pf*DXR, but comparable to fosmidomycin. This finding is of significant importance since *Ec*DXR was initially used as a surrogate for *Pf*DXR inhibition due to its difficult production and handling [100]. Kurz and coworkers concluded based on this series that *α*-phenyl derivatives with a free or *N*-methylated hydroxamic acid **(107a, b**) moiety are very promising derivatives for further drug development [93,100,101]. Introduction of electron-withdrawing chloro- and fluoro-substituents (**109, 110a, b**) led to excellent inhibitors of *Pf*DXR (IC_50_ = 3–4 nM), while electron-donating groups (**111**) decreased activity (Figure 24). Derivative **110b** was furthermore a potent inhibitor of *Pf*Dd2 in vitro (IC_50_ = 40 nM).

Interestingly, the in vivo efficacy of the difluorophenyl derivative **110a** (application of 80 mg/kg i.p. for 5 days) in a *P. berghei* ANKA mouse model at day 5 post-infection was almost similar to fosmidomycin. Compounds **110a** and **110b** reduced the percentage of infected erythrocytes significantly (89% and 78%, respectively), but the effect on mice survival was less pronounced, and no curative antimalarial activity was observed [100].

**Figure 25:**
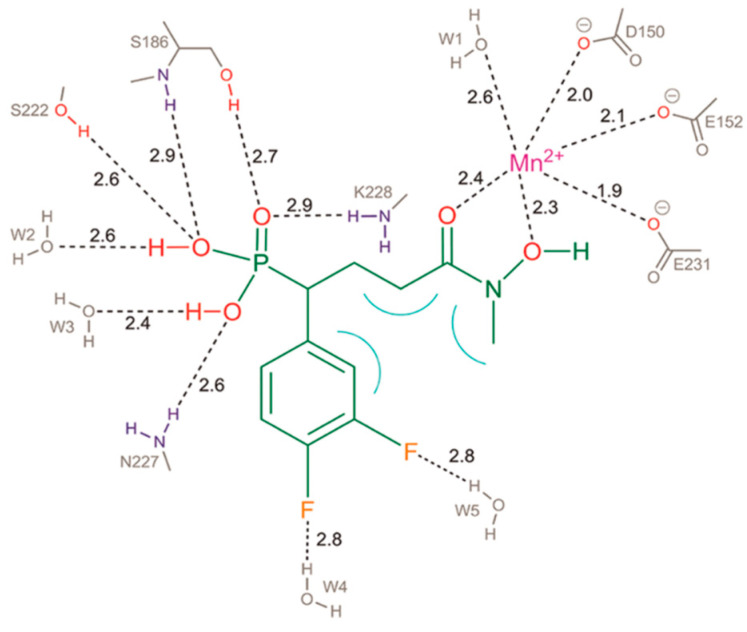
Schematic overview between **110b** (green) and the active site of *Ec*DXR. Intramolecular van der Waals interactions (light blue) between the *N*-methyl group with the difluorophenyl ring and the linker atoms of **110b**. Distances are in Å [100]. Reprinted with permission from *J. Med. Chem.* 2011, 54, 6796–6802 [100]. Copyright 2022 American Chemical Society.

Moreover, the X-ray crystal structure of *Ec*DXR in complex with fosmidomycin and Mn^2+^ (Figure 25) revealed that fosmidomycin fits perfectly into the closed conformation of the catalytic site, whereas the difluorophenyl ring of **110b** would clash with the flexible loop region and therefore binds to the open conformation of the catalytic site.

#### 4.3.5. Reverse α-Substituted Oxa, Thia and Aza Analogs

**Figure 26:**
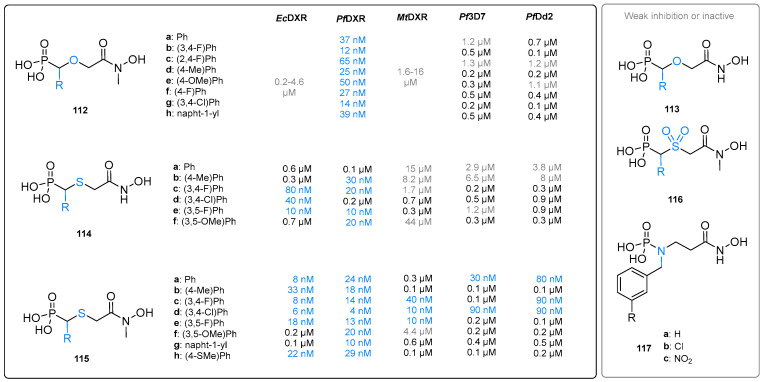
Antibacterial and antiplasmodial activity of thia (**112+113**), oxa (**114+115**), sulfone (**116**) and aza (**117**) analogs.

Based on the promising *α*-phenyl-substituted reverse carba analogs, Kurz and coworkers developed bioisosteric *α*-substituted *β*-thia and *β*-oxa analogs (**112**–**115**, Figure 26) [93,138,142,143]. Compounds **112**–**115** were tested against *Pf, Mt* and *Ec*DXR enzymes as well as in antiplasmodial growth assays towards *Pf*Dd2 and *Pf*3D7. The *β*-oxa analogs with an unsubstituted hydroxamic acid group (**113**) were at least 2 orders of magnitude less potent than their *N*-methylated analogs **112a**–**h** in all DXR enzymes and *Pf* growth assays. Derivatives **112a**–**h** were good to excellent inhibitors of *Pf*DXR (IC_50_ = 12–65 nM), but were less efficient in *Pf* growth assays (IC_50_ = 0.2 μM–1.3 μM). These results indicated that the *N*-methylation of the hydroxamic acid moiety is often beneficial for potent antiplasmodial in vitro activity of *α*-phenyl-substituted reverse analogs. The positive impact of *N*-methyl substitution was later confirmed for the *β*-thia-analogs (**115a**–**h**) as the non-methylated derivatives **114a**–**f** were in general less efficient compared to the *N*-methyl-substituted analogs. Analysis of the crystal structures of *N*-methylated derivatives showed, that the methyl group forms beneficial van der Waals interactions [100,138].

Furthermore, carba, oxa and thia analogs showed potent inhibitory activity towards the DXRs of *E. coli*, *P. falciparum* and *M. tuberculosis* (Figure 17 and Figure 26). The thia-analogs **115a**–**h** were more active against *Ec*DXR and *Mt*DXR than the carba analogs (**107b**–**111b**, Figure 24) while the carba derivatives displayed the strongest activity against *Pf*DXR. The oxa analogs (**112a**–**h, 113**) demonstrated the weakest inhibitory activity against all tested DXR enzymes. 

The higher activity of the thia analogs was explained by the interaction of the large polarizable sulfur atom with the highly conserved Met298 of the flexible loop. In oxa analogs, the considerably smaller electronegative oxygen atom would lead to a repulsive effect, while the carbon atom does not interact with the enzyme [138]. Kunfermann et al. identified the remarkable enantioselectivity of thia analog **115a** towards *Pf*DXR [138]. The highly active *S*-(+)-enantiomer of **115a** gave a IC_50_ of 9 nM, whereas the *R*-(−) enantiomer was virtually inactive with an IC_50_ of >10 μM. This was confirmed by the co-crystallization of the *S*-(+)-isomer of **115a** with *Pf*DXR. In addition, the *α*-3,4-dichlorophenyl-substituted derivative **115d** showed excellent inhibition of all tested DXR enzymes (*Ec, Mt, Pf*) with excellent IC_50_ values of 5-10 nM. In a later publication, the thioethers were oxidized to their corresponding sulfones with the general structure **116** (Figure 26). The sulfone derivatives were 2–3 orders of magnitude weaker inhibitors than their corresponding thioethers in respect to the three enzyme orthologs. The majority of the sulfone derivatives showed no significant inhibition of *Pf*3D7 growth in vitro. One exception is the α-3,5-dimethoxy substituted sulfone derivative (structure not shown), which displayed at least moderate antiplasmodial in vitro activity [143]. Adeyemi et al. recently synthesized a series of *α*-benzyl analogs (**117a**–**c**, Figure 26), where the benzyl residues were attached to a nitrogen atom instead of the *sp*^3^-hybridized *α*-carbon atom of the propyl linker, resulting in phosphoramidate analogs of reverse FR900098. The *α*-benzyl derivatives (**117a**–**c**) were non-cytotoxic to the mammalian cells, but only weakly active or inactive against *P. falciparum*. Docking studies suggested that the benzyl substituent would not fit into the substrate binding site of *Pf*DXR due to its size and conformational constraint [144].

#### 4.3.6. β- and γ-Substituted Analogs

**Figure 27:**
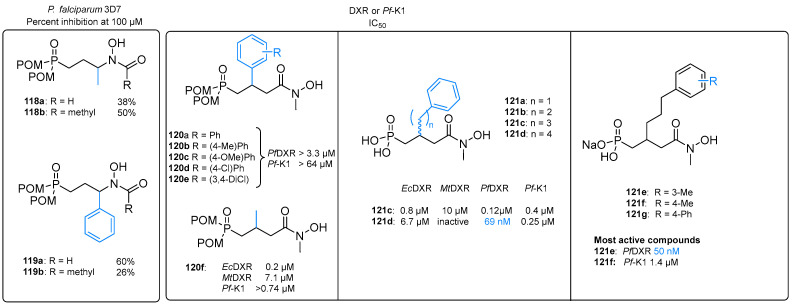
Antiplasmodial activity of β- and γ-substituted analogs (**118***–***121**).

Compared to *α*-substituted DXR inhibitors, there are fewer *β*- and *γ*-substituted DXR inhibitors available due to their more difficult synthetic accessibility. Earlier in the development of fosmidomycin analogs, Geffken and coworkers synthesized some moderately to weakly active POM-prodrugs of **1** and **2** with *γ*-methyl and *γ*-phenyl substitution (**118**–**119**, Figure 27) [133]. While the POM-prodrug of **1** inhibited 100% growth of the *Pf*3D7 strain at 100 µM, the activity for *γ*-methyl (**118a, b**) and *γ*-phenyl (**119a, b**) derivatives dropped to or below 60% growth inhibition. The weak antiplasmodial activity was attributed to the *γ*-methyl and *γ*-phenyl residues which could interfere with the interaction between the hydroxamic acid moiety and the divalent cation in the active site of DXR [133].

Van Calenbergh and coworkers [145] introduced aryl (**120a**–**e**, Figure 27), alkyl (**120f**), and aryl alkyl substituents (**121a**–**d**) at the *β*-position of lead structure **4** (Figure 9). 

If a *β*-aryl moiety (**120a**–**e**) is directly attached to the carbon linker, the derivatives are weak to poor inhibitors of *Ec, Pf* and *Mt*DXR. The reduction in inhibitory activity was lower in the *β*-methyl substituted analog (**120f**), but still significant. A small activity improvement was observed for inhibitors with arylalkyl residues in the *β*-position (**121a**–**d**). For example, compound **121c** with a phenyl-propyl residue inhibited *Ec*DXR and *Pf*DXR with IC_50_ values of 0.8 and 0.1 µM, respectively. In contrast, the *β*-phenyl-butyl derivative **121d** was more active against *Pf*DXR with an IC_50_ of 69 nM, surpassing the inhibitory activity of fosmidomycin. However, **121d** was less active towards *Ec*DXR and inactive against *Mt*DXR.

X-ray structures of **121c** and **121d** in complex with *Pf*DXR revealed that the longer, more flexible phenyl alkyl residues led to different flap structures in the case of *Pf*DXR. The *β*-phenyl residues of these compounds are in a boomerang shape and able to interact with their own *N*-methylated hydroxamic acid moiety as well as with Trp296 through an acyl-group-to-ring interaction. Additionally, the X-ray structures revealed that the *R*-enantiomer is primarily bound to the enzyme. 

Expanding their prior work, van Calenbergh and coworkers synthesized a series of *β*-arylpropyl derivatives (**121e**–**g**, Figure 27) bearing various substituted phenyl moieties [146]. The introduction of a methyl group in the 3-position in the case of **121e** improved inhibitory activity to 50 nM against *Ec*DXR compared to the unsubstituted phenyl ring (**121c**). An improvement of *Pf*-K1 inhibition was realized by the introduction of a methyl group in the 4-position of the phenyl ring (**121f**), leading to an IC_50_ value of 1.4 µM. The replacement of the phenyl moiety with a biphenyl substituent (**121g**) slightly decreased inhibitory activity against *Pf*DXR (IC_50_ = 1.6 µM) while significantly reducing inhibitory activity against *Pf*-K1 (IC_50_ > 64 µM). X-ray structure analysis of **121e** and other members of this series revealed that upon inhibitor binding the flap covering the active site was disordered resulting in key interactions of Trp296 with **1** and **2** being no longer possible.

## 5. Phosphonic Acid Isosteres and Bioisosteres

**Figure 28:**
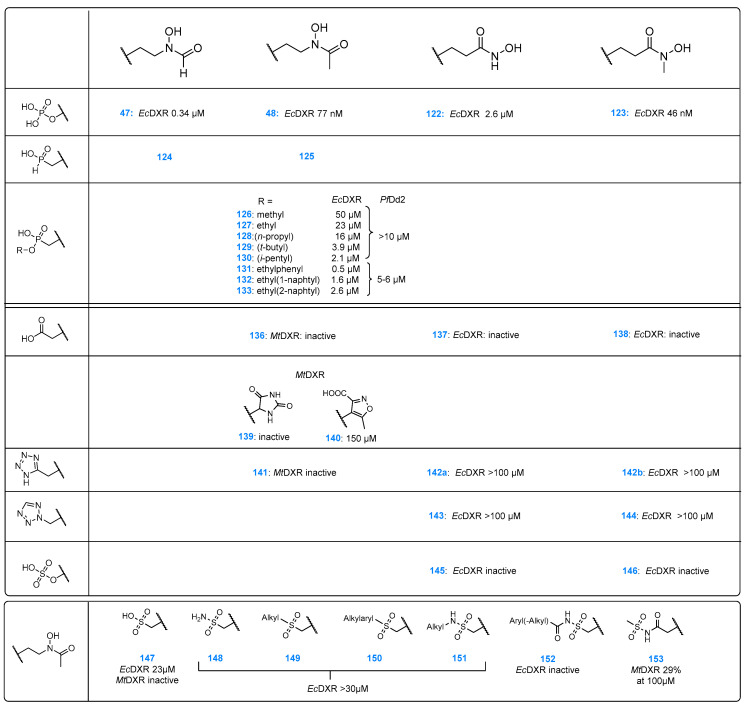
Biological data of phosphonic acid isosteres. Light blue: hydroxamate moiety. Grey: hydroxamic acid moiety. a: R = H. b: R = methyl. n.d. = not determined. Fosmidomycin (**1**) as a reference compound.

Fosfoxacin (**47**), first isolated from *P. fluorescens* in 1990 [122], is the phosphate bioisoster of fosmidomycin (Figure 15). In 2006, fosfoxacin (**47**) and its acetylated congener **48** (Figure 15) were first synthesized by Woo et al. and identified as more potent inhibitors of *Synechocystis* DXR than **1** (K_i_ **47** = 19 nM and K_i_ **48** = 2 nM vs. K_i_ **1** = 57 nM, data not shown in Figure 28) [98]. Munier et al. [147] continued investigating the replacement of the phosphonic acid moiety of **1**–**4** (Figure 9) by a phosphate moiety and evaluated the antibacterial efficacy of bioisosteres **47**–**48** and **122**–**123** against DXRs of *E. coli* and *M. smegmatis* (Figure 28, data for *M. smegmatis* not presented). The organic phosphates **123** (IC_50_ = 46 nM) and **48** (IC_50_ = 77 nM) inhibited *Ec*DXR in a similar range as fosmidomycin (IC_50_ = 42 nM) but are still 12–20-fold weaker inhibitors than **2** (IC_50_ = 4 nM, Figure 1). Surprisingly, the formyl analog **47** and the non-methylated hydroxamic acid analog **122** are only moderate to weak inhibitors of *Ec*DXR (IC_50_ = 0.34 µM and 2.6 µM, respectively). Furthermore, **47**–**48** and **122**–**123** were tested in bacterial growth assays but were less effective than **1** and **2** (Figure 1) in *Ec* and a fosmidomycin-resistant *Ec* strain (FosR). In general, it was unexpected that all phosphonic acid derivatives were more potent than their phosphate analogs because phosphate derivatives should fit better into the phosphate-binding site of DXR [147,148].

Fujisawa Pharmaceutical Co., Ltd., performed one of the earliest attempts to replace the phosphonic acid with a phosphinic acid group (**124**–**125**, Figure 28). However, both phosphinic acid analogs (**124**–**125**) were less active against selected Gram-negative bacteria than **1** and **2 [118]**.

Perruchon et al. [134] retained the phosphonic acid group and synthesized biologically stable monoalkyl phosphonates (**126**–**133**, Figure 28) that are not rapidly cleaved by phosphatases [149,150]. To further investigate the possible presence of an extended binding region, compounds (**131**–**133**, Figure 28) were synthesized [134]. Indeed, the activity against *Ec*DXR increased with the chain length of the alkyl residue (IC_50_ = 50 µM for **126** with methyl group vs. IC_50_ = 2.1 µM for **130** with isopentyl group), suggesting the presence of the proposed lipophilic binding region. However, the alkyl monoesters showed no measurable antiplasmodial activity (IC_50_ > 10 µM) with the exception of the arylethyl monoesters (**131**–**133**), which showed weak inhibitory activities against *Pf*Dd2 strains (IC_50_ = 5–6 µM). In summary, it was demonstrated that both hydroxy groups of the phosphonic acid moiety are mandatory for potent inhibitory activity and all synthesized derivatives displayed low in vitro inhibitory activity against *Pf*Dd2 and *Ec*DXR.

Furthermore, different research groups replaced the phosphonic acid group with isosteric groups, which are summarized in the second half of Figure 28. Derivatives with a carboxylic acid moiety (**136**–**138**, Figure 28) were inactive against *Synechocystis*, *Ec* or *Mt* DXR [98,99,151]. A possible explanation for the loss of inhibitory activity resulting from the phosphonic acid replacement is the planar geometry of a carboxylic acid moiety compared to the pyramidal geometry of the phosphonic acid group. 

In 2011, Andaloussi et al. [104] tried to identify less polar *Mt*DXR inhibitors, which can penetrate the highly lipophilic cell wall by replacing the phosphonate with carboxylic acid bioisosters (**139**–**141**, Figure 28). Only the isoxazole carboxylic acid analog (**140**) showed negligible *Mt*DXR inhibition with an IC_50_ of 150 µM (IC_50_ **1** = 0.08 µM), while the hydantoin (**139**) and C-substituted tetrazoles (**141**) showed no inhibition [104]. Nguyen-Trung et al. [152] developed analogous *C*- and *N*-substituted tetrazole derivatives (**142**–**144**, Figure 28), which demonstrated no inhibitory activity towards *Ec*DXR. The loss of activity was explained by the planar rigid structure of the tetrazole, which could disrupt several potential interactions between the heterocycle and various amino acids in the active site of DXR. The authors hypothesized that **142**–**144** were unable to occupy the hydroxamate and phosphonic acid binding sites simultaneously.

In addition, several working groups studied the importance of the phosphonic acid pharmacophore by replacing this moiety with charged and uncharged sulfur-containing functional groups.

The mono alkyl sulfate analogs (**145**–**146**), sulfonic acid (**147**) and sulfamate analog **148** (Figure 28) were weak or inactive against *Ec*DXR [98,99,151], likely due to their different abilities to form hydrogen-bond interactions compared to the phosphonic acid moiety [153]. Perruchon et al. [134] synthesized derivatives with polar, but uncharged, sulfone or sulfonamide moieties (**149**–**151**, Figure 28). Both groups contain two oxygen atoms which could act as hydrogen bond acceptors and form hydrogen-bond networks with Ser186, Ser222, Asn227 and Lys228, akin to the interactions of the phosphonic acid group. Furthermore, Perruchon et al. analyzed the surface of the phosphonic acid binding site and identified a small sub-pocket that eventually permits the attachment of small additional residues. The sulfone derivatives with small alkyl (**149**) and arylalkyl moieties (**150**) can only act as hydrogen bond acceptors, while the N-H moiety of the sulfonamide (**150**) might form hydrogen bonds with Ser186 and Ser222 due to their side-chain flexibility, which allows rotational and conformational changes. However, no derivative showed inhibitory activity against *Ec*DXR (Figure 28). In 2015, Gadakh et al. [154] continued the efforts regarding possible replacements for the phosphonic acid pharmacophore by modifying the unsubstituted sulfonamide moiety. The authors synthesized four *N*-acylated sulfonamides with methyl, phenyl, benzyl and phenylethyl residues (**151**, Figure 28). Even though molecular modelling results indicated the occupation of a larger hydrophobic pocket like for the arylethyl esters (**131**–**133**), the compounds with an *N*-acyl sulfonamide moiety (**152**, Figure 28) were completely inactive against *Ec*DXR. At least, 29% inhibition of *Mt*DXR was observed for the *N*-(methylsulfonyl)amide (**153**, Figure 28) at a concentration of 100 µM. A possible explanation for the lack of activity of compounds **151**–**153** could be the strong delocalization of the negative charge weakening the hydrogen bond network in the phosphate-binding site.

In summary, derivatives with sulfamate or *N*-substituted tetrazole derivatives as neutral molecules, as well as carboxylic acid, sulfonate and *C*-substituted tetrazole derivatives as monoprotonic acids and mono- and diesters of the phosphonic acid moiety do not inhibit DXR enzymes. 

The data presented underscore the importance of two negatively charged groups which are present in the (monoalkyl)phosphate and the phosphonate moiety. However, differences between phosphonate and phosphate-based inhibitors (**47**–**48** and **122**–**123**) observed in antimicrobial growth assays could be related to the cell wall and/or membrane penetration and chemical/metabolic stability. Interestingly, the fact that phosphate-based derivatives (**47**–**48**) inhibited *Ec* growth indicated that highly hydrophilic phosphates can penetrate into cells, likely via the glycerol 3-phosphate transporter (GlpT) and/or the hexose 6-phosphate (UhpT) transporter [155,156,157]. This hypothesis is supported by the lack of inhibition observed in a fosmidomycin-resistant *Ec* strain in which GlpT/UhpT transporters are not active [158]. Besides this, the metabolic instability of organic phosphates due to their cleavage and inactivation by phosphatases is well described and can contribute to the different activities of phosphates compared to the phosphonic acid-based inhibitors [98]. Consequently, further investigation by several groups concentrated on the synthesis of phosphonic acid prodrugs and derivatives.

## 6. Conclusions Regarding Structure–Activity Relationship 

**Figure 29:**
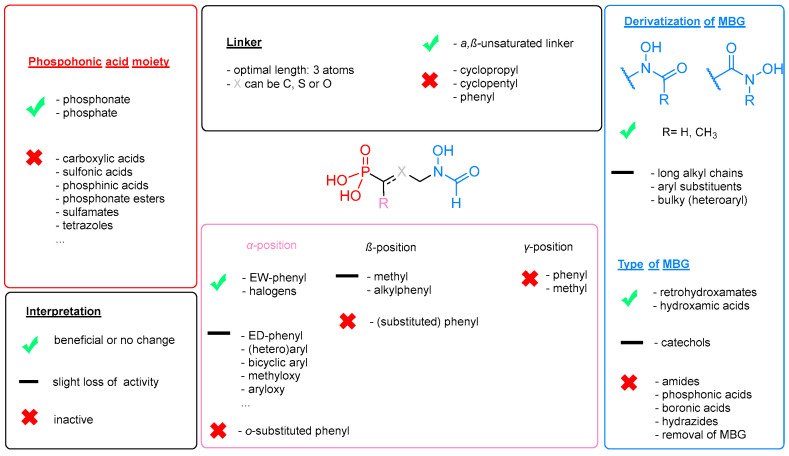
Structure–activity relationship (SAR) of fosmidomycin derivatives. EW = electron withdrawing, ED = electron donating, o = ortho, MBG = metal binding group.

The hydroxamate and retro-hydroxamate moiety are thus far the only suitable MBGs that result in potent DXR inhibitory activity. Inhibitors with hydroxamate and retro-hydroxamate MBGs showed comparable activity and no enhanced selectivity for specific bacteria or parasites. All analogs with (bio)isosteric replacements for these groups were only weakly active or inactive (Figure 29). The tight structure–activity relationship was also demonstrated for the phosphonic acid moiety. Only the naturally occurring monoalkylphosphate fosfoxacin (**47**) and the phosphate analogs **48**, and **122**–**123** (Figure 15) showed comparable or slightly superior activity against DXRs compared with fosmidomycin. All other tested di- and monoprotonic acids (carboxylates, sulfonates, phosphonic acids) as well as non-dissociating moieties (sulfamates, *N*-substituted tetrazoles) showed no DXR inhibition. Furthermore, it has been demonstrated that both hydroxy groups of the phosphonic acid moiety are essential for potent inhibitory activity.

To optimize the linker, several structurally diverse inhibitors were synthesized: The optimal linker consists of three atoms being classified as α-, β- and γ-atoms, which in most inhibitors are carbon atoms. Nitrogen atoms in the α-position and oxygen atoms in the γ-position were not tolerated, while β-oxa analogs were partly tolerated. However, a sulfur atom in the β-position of the linker (**115**, Figure 26), led to the most potent known inhibitors and showed increased selectivity for *Mt*DXR. The majority of conformationally restricted linkers were not tolerated. Two exceptions are a trans-configured cyclopropyl linker (**55**, Figure 16), which displayed similar activity as fosmidomycin (**1**), and an unsaturated propenyl linker as in FR32863 (**IV**, Figure 2), which was active in the nanomolar range. 

In the α position electron-withdrawing chlorine and fluorine atoms increased activity and the acidity of the phosphonate moiety. Furthermore, *α*-phenyl substituents, especially with electron-withdrawing residues in *para*- and *meta*-position such as fluorine (**110a, b**, Figure 24 and **114c, 115c**, Figure 26), were beneficial. *β*- and *γ*- substituted analogs are scarce and these types of modifications are mostly not well tolerated. 

Despite the promising anti-infective activity of DXR inhibitors in vitro, to date, none of the inhibitors exhibited significant curative antimalarial in vivo activity in infectious mouse models. However, among emerging diverse inhibitors, some derivatives showed excellent DXR enzyme inhibition in the low nanomolar range.

## 7. Prodrugs of Fosmidomycin and Its Analogs

Phosphonic acid groups in drugs and drug candidates are often associated with unfavorable and challenging physicochemical and ADME properties. Despite their stability towards phosphatases, the membrane permeability and subsequent cellular uptake of small molecules containing phosphonic acid groups are often insufficient [159,160,161]. The poor membrane permeability of phosphonic acids is due to their anionic nature at physiological pH. To mask the anionic structure and to overcome the limitation described above, various types of phosphonic acid prodrugs were and are still under development. The overall goal of this prodrug concept is to enable efficient oral administration of phosphonic acid-based small molecules.

Particularly challenging organisms include *Mt* due to its highly lipophilic mycolic acid-containing cell wall, and parasites with apicoplasts, in which the DXR enzyme is located. In *Pf* erythrocyte models, prodrugs need to pass seven membranes and the exact compartment of bioactivation is not known [36]. To date, all in vivo studies with DXR inhibitors have been conducted with mice infected with *Plasmodia*. 

To mask the highly polar phosphonic acid moiety of fosmidomycin and its analogs and to achieve in vivo efficacy against malaria, established prodrug concepts commonly used for antiviral drugs were employed. These concepts include lipophilic phosphonate esters, e.g., tenofovir disoproxil (Viread, 2001) [162] containing a bis-POC (isopropyloxycarbonyloxymethyl) moiety and adefovir dipivoxil (Hepsera, 2002) [163] with a bis-POM (pivaloyloxymethyl) moiety, aryloxyphosphoramidate (e.g., remdesivir, sofosbuvir) [161,164,165,166,167], aryloxyphosphonamidate prodrugs (e.g., tenofovir alafenamide) [162,168,169] phosphobisamidates [170], cyclic esters and monoalkylphosphonates [163].

### 7.1. Lipophilic Phosphonic Acid Esters

**Figure 30:**
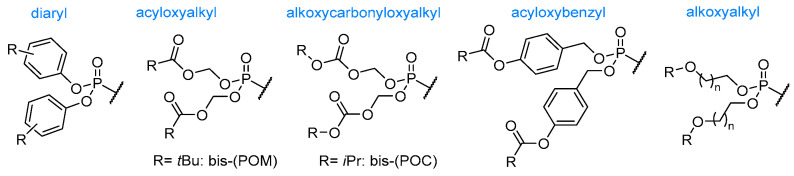
Lipophilic phosphonate prodrugs used for fosmidomycin and its analogs.

Aiming to overcome the poor permeability and absorption as well as the relatively short half-life time of fosmidomycin and FR900098 (Figure 1) [61,62], several groups developed phosphonic acid prodrugs to increase oral bioavailability and in vivo efficacy. Most prodrug moieties increase the lipophilicity of the phosphonic acid group. The different structure types of lipophilic prodrugs presented in the following chapters are summarized in Figure 30.

#### 7.1.1. Ester Prodrugs of Fosmidomycin and FR900098

**Figure 31:**
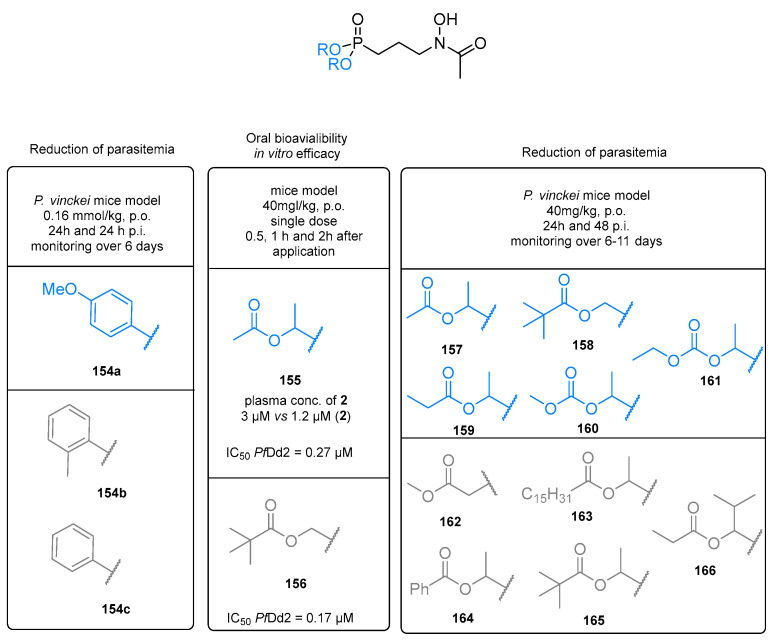
Results of in vivo studies of lipophilic FR900098 (**2**) esters. Blue indicates improvement and grey reduction of efficacy/plasma concentration of the prodrug compared to parent compound **2**. p.o. = per os, p.i. = postinfection.

In 2001, the first in vivo studies with FR900098 (**2**, Figure 1) and its diaryl ester prodrugs were conducted by Wiesner et al. (**154a**–**c**, Figure 31). While a significant increase in the in vivo antimalarial efficacy for the bis(4-methoxyphenyl) diester prodrug (**154c**) was observed, no curative properties have been detected [171].

Due to toxicity concerns for the liberated phenols upon bioconversion, alternative lipophilic phosphonic acid derivatives such as acyloxyalkyl and alkoxycarbonyloxyethyl esters (Figure 30) of FR900098 (**2**) have been synthesized by the Schlitzer group. These derivatives were designed to reduce toxicity while increasing bioavailability and antimalarial in vivo activity [172,173,174]. Initial investigations documented an improved in vitro antiplasmodial activity of the FR900098-prodrug **155** against *Pf*3D7 and Dd2 strains as well as increased oral bioavailability. To prove the bioconversion of **155** and verify oral bioavailability, 40 mg/kg of ester prodrug **155** and **2** were orally administered to mice. **2** showed a plasma concentration of 1.2 µM after 30 min while application of **155** led to an improved plasma concentration of 3 µM of **2** after cleavage of the prodrug moiety by plasma esterases. The plasma levels of **155** and **2** were below the detection limit 2 h after application underlining the poor pharmacokinetic properties of **2** and its prodrugs [173].

Following up on this concept, the Schlitzer group synthesized further bis[(acyloxy)alkyl] (**157**–**159, 162**–**166**, Figure 31) and bis[(alkoxycarbonyloxy) ethyl] ester (**160**–**161**) prodrugs of **2** and tested them in a *P. vinckei* mouse model over 6 to 11 days. Even though the prodrug **158** was more active than its parent compound **2**, the formation of formaldehyde from the dioxymethylene group was classified as unfavorable. In contrast, prodrugs containing a 1,1-dioxyethylene group (**157, 159**–**161, 163**–**165**) released less problematic acetaldehyde during bioactivation. 

The most promising derivative **157** was tested in vivo and compared with FR900098. 10 mg/kg of **157** showed the same activity as 40 mg/kg of FR900098, while higher dosages up to 40 mg/kg of **157** exceeded the efficacy of FR900098 after 8 days [174]. Although the in vivo efficacy of **2** has been improved through the development of prodrugs, further improvements are necessary in order to provide candidates with curative properties.

**Figure 32:**
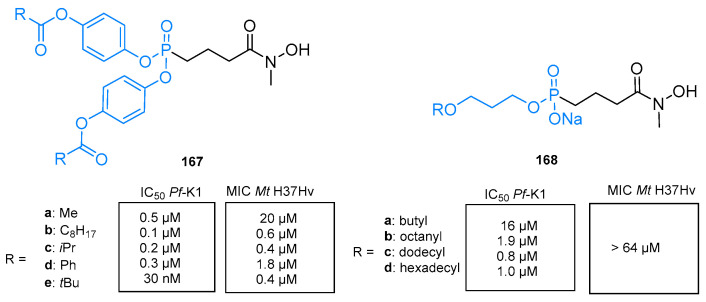
Antiplasmodial and antimycobacterial activity of acyloxybenzyl (**157**) and alkoxyalkyl phosphonate ester prodrugs (**158**).

**Figure 33:**
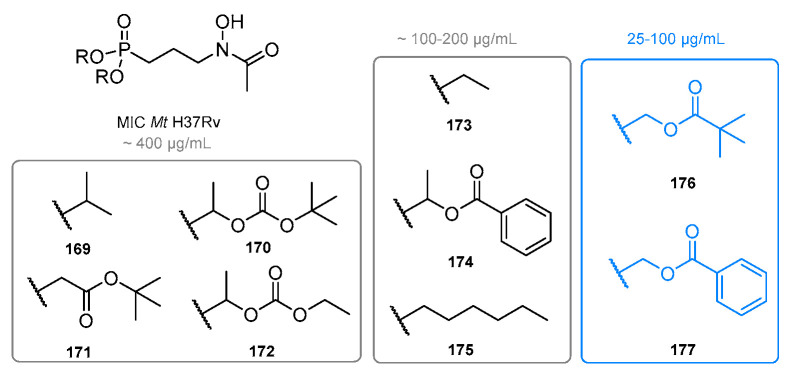
Inhibitory activity of different FR900098 prodrugs (**169**–**177**) against *M. tuberculosis* H37Rv in growth assay.

In 2017, Courtens et al. [175] described acyloxybenzyl and alkoxyalkyl phosphonate ester prodrugs of the reverse FR900098 **(167**–**168**, Figure 32). The acyloxybenzyl prodrugs **167a**–**e** surpassed the inhibitory activity of fosmidomycin against *Pf*-K1 (IC_50_ (**1**) = 1.7 µM) and *Mt* H37Ra strains (IC_50_ (**1**) > 64 µM). Among the tested compounds, the acetyl ester **167a** was the weakest inhibitor, while **167c** and **167e** were highly active derivatives (IC_50_ = 0.4 µM) against *Mt*. However, prodrugs **167a**–**e** showed cytotoxic effects in the MRC-5 fibroblast cell line. In contrast to alkyloxymethyl prodrugs, acyloxybenzyl prodrugs release the reactive electrophile quinone methide during bioactivation, which could be the reason for the observed cytotoxicity [175]. The synthesized monoalkoxyalkyl phosphonates (**168a**–**d**) were not active against *Mt*, but the antiplasmodial activity of dodecyl **168c** and hexadecyl analog **168d** exceeded the efficacy of fosmidomycin. The authors hypothesized that the long alkyl chains could improve passive diffusion. The Dowd group elucidated the activity of phosphonate ester as potential prodrugs of **2** against a panel of Gram-positive (*B. anthracis*, *E. faecalis*, *S. aureus* (MSSA and MRSA)) and Gram-negative bacteria (*Acinetobacter* and *E. coli*) as well as *Mt* (Figure 33) [42]. Consistent with previous results with dialkyl ester prodrugs, the prodrugs **169, 173** and **175** showed weak or reduced activity against these bacteria (for results of all tested bacteria, see Uh et al. [42]), which was expected due to insufficient bioactivation of small aliphatic dialkylphosphonates.

The bis-POM ester (**176**) (MIC = 50–100 µg/mL) and the bis-[benzoyloxymethyl] ester (**177**) (MIC = 25–100 µg/mL) were moderately active against *Mt* H37Rv. In general, the antimycobacterial activity increases with the size of the prodrug moiety [42]. In *Mt*, the uptake of hydrophilic DXR inhibitors does not occur via the GlpT transporter due to its absence. Consequently, the effectiveness of the prodrugs **176** and **177** mainly relies on their lipophilicity, which enables penetration of the membranes by passive diffusion. Moreover, it was hypothesized that prodrugs **176** and **177** circumvent resistance caused by *glpT* mutations observed in bacteria where uptake of DXR inhibitors is reliant on the GlpT. The second observation made in this study is that ester prodrugs of primary alcohols (**176, 177**) are more active than esters prodrugs of secondary alcohols (**170**, **172, 174**). It was hypothesized that cellular esterases are unable or only partially able to hydrolyze substrates with a substituted acyl moiety [42,176].

**Figure 34:**
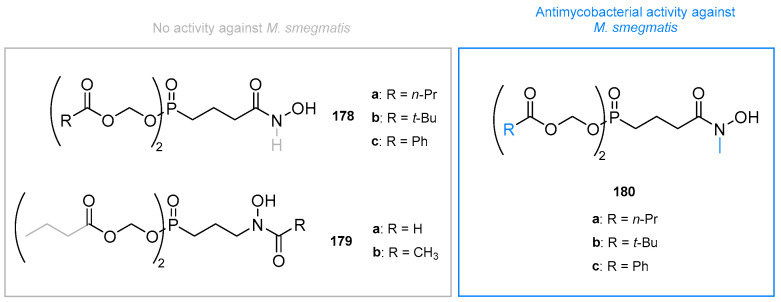
Antimycobacterial activity of prodrugs (**178***–***180**) against M. smegmatis determined by paper disc diffusion method.

In 2012, Ponaire et al. reported further acyloxymethyl phosphonate prodrugs of **3** and **4** (**178**–**180**, Figure 34) and tested their activity in an *M. smegmatis* growth assay using a paper disc diffusion method with a concentration of 400 nmol/disk. (Figure 34) [177]. The phosphonic acids **1** and **2** inhibited the growth of *M. smegmatis,* whereas the prodrugs **178a**–**c** showed no growth inhibition. Prodrugs of *N*-methylated DXR inhibitors (**180a**–**c**) moderately inhibited mycobacterial growth. The *n*-propionyloxymethyl prodrug **180a** was more active than the lipophilic POM (**180b**) and benzoyloxymethyl (**180c**) prodrugs [178]. This suggested that the uptake of bulky and rigid prodrugs might be restricted. As the most active prodrug **180a** contained an *n*-propionyloxymethyl moiety, the *n*-propionyloxymethyl prodrugs of **1** and **2** were prepared (**179a, b**). However, **179a, b** demonstrated no growth inhibitory effects. 

#### 7.1.2. Ester Prodrugs of Fosmidomycin Analogs

**Figure 35:**
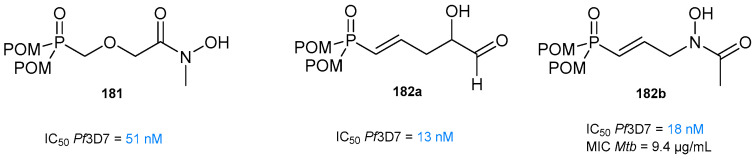
Structure and antiplasmodial activity of prodrugs **181** and **182**.

Haemers et al. synthesized the *β*-oxa isostere of **4** (**54**, Figure 15) and the corresponding POM-prodrug (**181**, Figure 35), which showed a 4-fold improvement in in vitro efficacy against *Pf*3D7 compared to the parent compound **54** [123]. The Dowd group designed and synthesized POM prodrugs (**182a, b**) of the natural product FR32863 (**IV**) and the acetylated analog **42** (Figure 35). Both prodrugs (**182a, b**) Prodrug **182a** was identified as a potent inhibitor of *Pf* growth with an IC_50_ of 13 nM [179].

The POM prodrug **182a** was further evaluated for in vivo efficacy in a *P. berghei*-infected mouse model of malaria, where groups of mice were infected with luciferase-based blood-stage *P. berghei* ANKA (PbA). Prodrug **182a** reduced parasitemia in the mice treated with a dose of 20 mg/kg for 5 days. Moreover, **182a** was well tolerated and showed no evidence of cytotoxicity in vitro [121]. The POM-prodrug **182b** was only tested in vitro in an *Mt* growth assay and compared to parent compound **45** (Figure 14) increased the MIC from >200 μg/mL to 9.4 μg/mL [119].

**Figure 36:**
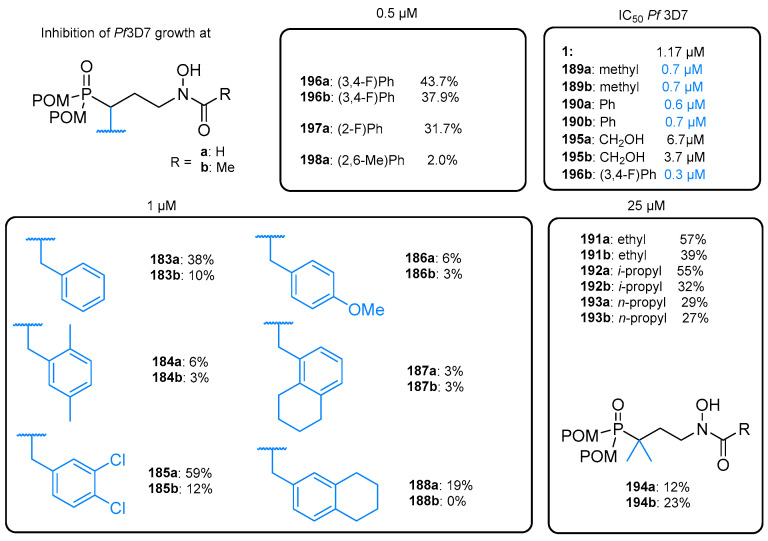
Antiplasmodial activity of α-benzyl and α-alkyl analogs **183***–***198**. Derivatives were screened for their inhibition of *P. falciparum* 3D7 growth at 0.5, 1 and 25 µM.

In 2006, Schlüter et al. published a series of *α*-benzylated POM-prodrugs shown in Figure 36. These prodrugs contain benzyl (**183**), 2,5-dimethyl-benzyl (**184**), 3,4-dichlorobenzyl (**185**), 4-methoxybenzyl (**186**) and (5,6,7,8)tetrahydronaphthalen-eylmethyl (THN) (**187**–**188**) substituents in the *α-*position of the propyl linker (Figure 36). While the prodrugs with a 3,4-dichlorobenzyl (**185**) moiety retained most of their antiplasmodial activity (59% inhibition at 1 µM) compared to **1** and **2** (32% and 71% at 1 µM), all other bulky substituents at the phenyl moiety drastically reduced antiplasmodial potency [131]. Schlüter et al. expanded the SAR analysis of the substitution pattern in the *α-*position by comparing the antiplasmodial activities of a number of *α*-alkyl-substituted (methyl, dimethyl, ethyl, *n*-propyl and *i*-propyl) inhibitors (**189, 191**–**194**) with POM-prodrugs of **1** and **2** (Figure 36) [131] The *α*-methyl-substituted prodrugs (**189a, b**) exerted significant antiplasmodial activity (IC_50_ = 0.7 µM), while longer alkyl chains led to a considerable loss of antimalarial activity (less than 50% *Pf* growth inhibition at 25 µM, data and structures not shown). The *α-*phenyl derivatives (**190a, b**) exhibited similar antiplasmodial activity to (**189a, b**). Extending this concept, the *N*-acetylated and *N*-formylated derivatives with *α*-hydroxymethyl (**195a, b**) and fluorinated *α*-phenyl substituents (**196**–**198**) were prepared and evaluated. The *α*-hydroxymethyl moiety compromised the inhibitory effects and reduced activities of inhibitors with IC_50_ values of 6.7 µM for **195a** and 3.7 µM for **195b**. The presence of the *α*-2,6-dimethyl-phenyl group in **198a** led to reduced activity. In contrast, the introduction of an *α*-3,4-difluoro phenyl substituent (**196a**) increased the potency towards *Pf* compared to the analog bearing an unsubstituted phenyl ring (43.7% inhibition @ 0.5 µM for **196a** vs. 39% for **190a** with an *α*-phenyl substituent) [133].

**Figure 37:**
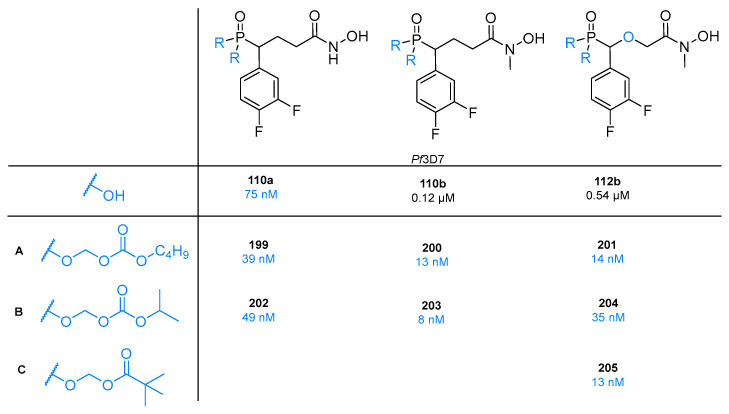
Antiplasmodial activity of parent compounds (**110a**, **110b**, **114b**) and prodrugs (**199***–***205**) against *Pf*3D7.

The Kurz group also synthesized phosphonate ester prodrugs of reverse *β*-oxa and carba analogs with a 3,4-difluorophenyl substituent in the *α*-position of the linker (**110a**, **110b** and **114b**, Figure 37). The phosphonic acid group of the inhibitors **110a**, **110b** and **114b** was masked with *n*-butyloxycarbonyloxymethyl (**A**), POC (**B**) and POM (**C**) prodrug moieties (Figure 37). All prodrugs of this series (**199**–**205**, Figure 37) were excellent to potent inhibitors of *Pf*3D7 and Dd2 with IC_50_ values between 8 and 49 nM and were more potent than their respective parent compounds (IC_50_ = 0.075–0.54 μM) [37]. Significant growth inhibition was achieved by the introduction of the POM prodrug moiety unit into the *β*-oxa analog **205** with an IC_50_ value of 13 nM against *Pf*3D7. **205** was more than 40-fold more active than the parent compound **114b** (IC_50_ value of 0.54 μM). The antiplasmodial activity of **114b** was further surpassed by the carba analog **203** with an excellent IC_50_ value of 8 nM. The inhibitors with an *n*-butyloxycarbonyloxymethyl prodrug unit (**A**) outperformed the inhibitors with a POC prodrug unit (**B**), which could be due to less steric hindrance during enzymatic or chemical cleavage. Notably, the *n*-butyloxycarbonyloxymethyl (**199**, **200**, **201**) and POM (**205**) prodrugs exhibited the same antiplasmodial potency (IC_50_ = 13–39 nM) in a whole-cell assay as their parent phosphonic acids **110a, b** and **114b** in a *Pf*DXR enzyme assay. Finally, it should be mentioned that, to date, no prodrugs of the highly active reverse thia analogs were synthesized, which could be an interesting strategy, especially for *Mt* drug research.

**Figure 38:**
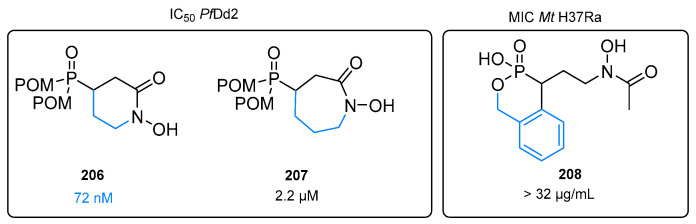
Inhibitory activity of rigidized prodrugs **206***–***208** against *Pf*Dd2 or *Mt* H37Ra.

Faísca Phillips et al. [180] combined several concepts that increased antiplasmodial activity and synthesized the rigidized 1-hydroxy-piperidin-2-one analogs **206** and 1-hydroxy-azepan-2-one **207** (Figure 38). In accordance with the design, the cyclic POM-prodrug **208** surpassed the activity of fosmidomycin, FR900098, and its POM-prodrug (IC_50_ = 72 nM) against *Pf*Dd2. 

An interesting example of an intramolecular cyclic phosphonate is the potential prodrug **208** synthesized by Andaloussi et al. (Figure 38) [128]. While the parent compound with a free phosphonic acid (**83**, Figure 18) showed 36% growth inhibition of *Mt*DXR at 100 µM, the cyclic ester was 3-fold less active and showed little activity. Both compounds lacked activity against *Mt* H37Ra. 

### 7.2. Double Prodrugs

**Figure 39:**
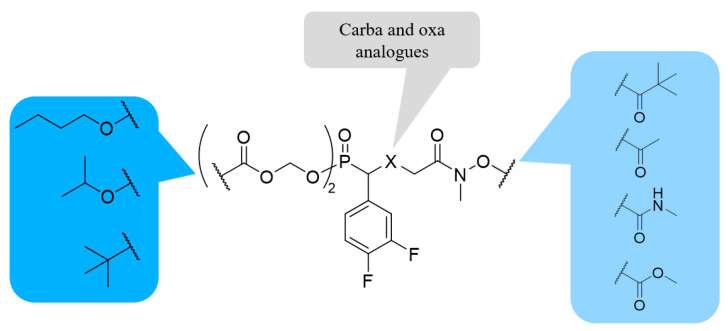
Schematic overview of reverse α-3,4-difluorophenyl-substituted β-oxa and carba analogs.

In 2012 and 2015 Kurz and collaborators also published the first double ester prodrugs of the reverse *α*-3,4-difluorophenyl-substituted DXR inhibitors (**108b** and **112b**, see Section 4.3.5) containing acyloxymethyl or alkoxycarbonyloxymethyl phosphonate prodrug moieties. [127] Since the penetration of several membranes is necessary to reach the target DXR in the plasmodial apicoplast, the hydroxy group of the hydroxamic acid moiety was also masked by acetate, pivalate, carbonate and carbamate (Figure 39) [37]. By masking the phosphonic acid and hydroxamate structures concurrently, the calculated log *p* values increased significantly (log *p* = −2.5 for **1** vs. log *p* > 2.5 for all double prodrugs) [37].

As expected, none of the double prodrugs inhibited *Pf*DXR. Surprisingly, despite the nanomolar antiplasmodial in vitro activity against *Pf*Dd2 (IC_50_ = 4–70 nM) and 3D7 (IC_50_ = 8–20 nM) no significant in vivo antimalarial activity in a *P. berghei* and a humanized SCID *P. falciparum* mouse model was observed.

**Figure 40:**
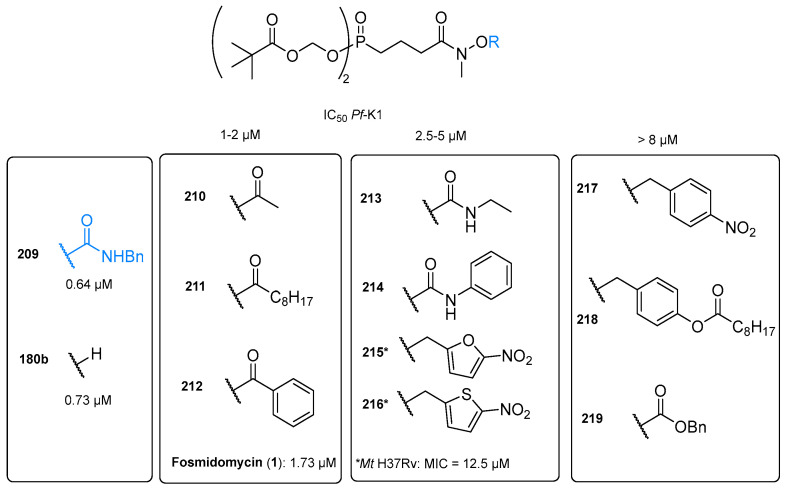
Antiplasmodial activity of double prodrugs **209***–***219** against asexual blood stages of *Pf*-K1 (IC_50_ in μM) and *Mt* H37Rv (MIC in μM).

By combining the concept of POM-phosphonate prodrugs with different hydroxamic acid prodrugs such as carboxylic acid esters, carbonates and carbamates, the Van Calenbergh group synthesized novel double prodrugs (**209**–**219**, Figure 40). The biological evaluation of this series was performed via whole-cell assays against *Pf*-K1 and *Mt* H37Rv or H37Ra [181]. Although, the majority of compounds (**209**–**214, 217**–**219**) did not inhibit *Mt* growth, prodrugs with a 2-nitrofuran (**215**) and 2-nitrothiophene (**216**) moiety were weak *Mt* H37Rv inhibitors with MIC of 12.5 μM. The authors hypothesized that **215** and **216** are bioreductive prodrugs. Unfortunately, significant cytotoxicity of **215** and **216** against MRC-5 fibroblasts was observed, diminishing their selectivity indices to approximately 2. The carbonate prodrug **219** and the nonanoyloxybenzyl ester prodrug **218** showed low activity against *Pf-*K1, while the ester prodrugs **210**–**212** were equipotent to fosmidomycin. The *N*-benzyl substituted carbamate prodrug **209** was the only retro-hydroxamate to surpass the activity of its parent compound **180b** (IC_50_ of **209** = 0.64 µM vs. IC_50_ of **180b** = 0.73 µM). To date, no in vivo data of these double prodrugs have been published. 

In conclusion, while the bioconversion of the POC- and POM-prodrug is well studied, further studies regarding the bioactivation of hydroxamate prodrugs in vitro and in vivo are required. 

### 7.3. Amino Acid Esters and Phosphonamidate Prodrugs

A third prodrug strategy, that is already well-studied and applied to improve pharmacokinetic properties in hepatitis and HIV therapies is the phosphonoamidate prodrug concept. Aryloxyphosphoramidate prodrugs that are currently in clinical use are sofosbuvir (Sovaldi, 2013) [166,167] and remdesivir (Veklury, 2020) [161] while tenofovir alafenamide (Vemlidy, 2015) [168] is the only aryloxyphosphonamidate in clinical use.

**Figure 41:**
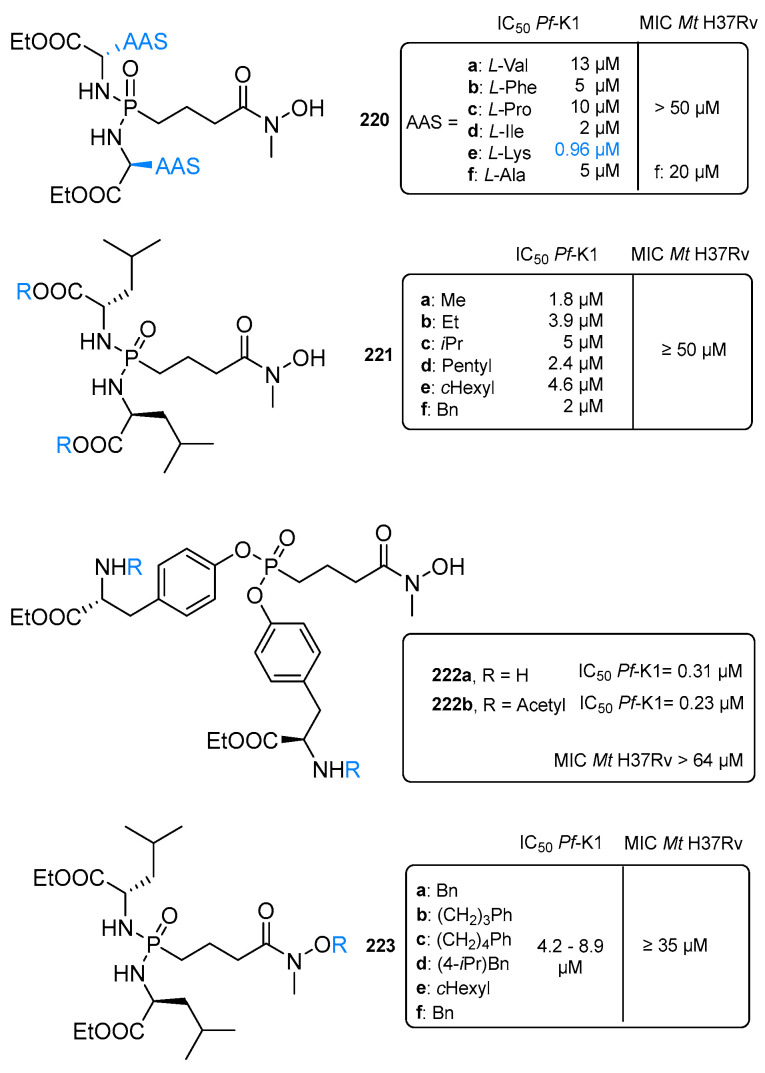
Biological data of amino acid-based prodrugs **220a**–**f**, **221a**–**f**, **222a**–**b**, and **223a**–**f** against asexual blood stages of *Pf*-K1 (IC_50_ in μM) and *Mt* H37Rv (MIC in μM). AAS = Amino acid side chain.

The Calenbergh group published two series of amino acid-based reverse N-methyl-fosmidomycin derivatives (**220***–***223**) and their in vitro inhibitory activity was tested against *Mt* H37Rv and asexual blood stages of *Pf*-K1 (Figure 41) [182,183]. In the first series, they focused solely on the conversion of the phosphonate moiety into bis-phosphonamidate prodrugs. These modifications were performed by varying amino acid residues (**220a**–**f**). Only the *L*-lysine-based bis-phosphonamidate prodrug (**220e**) showed submicromolar activity against *Pf* (IC_50_ = 0.96 µM), while the other derivatives showed activity similar to fosmidomycin. The *L*-tyrosine **222a** (IC_50_ = 0.23 µM) and *N*-acetyl-tyrosine **222b** (IC_50_ = 0.31 µM) prodrugs showed improved inhibition of *Pf*-K1 growth in comparison with fosmidomycin (IC_50_ = 1.73 µM). According to the authors, the likely protonation of **222a** at physiological pH did not appear to affect the uptake into red blood cells. Against the H37Rv wild-type strain of *Mt*, only the *L*-leucine **221b** and *L*-alanine **220f** based prodrugs exhibited weak activity with MIC values of 50 and 20 µM, respectively. The other derivatives did not show activity, which might be attributed to a lack of uptake. Furthermore, **220e** and **222b** were evaluated in a *P. berghei* malaria mouse model with a dose of 50 mg/kg applied intraperitoneally for 5 consecutive days. Compound **220e** failed to show a reduction of parasitemia post-infection, probably due to chemical/metabolic stability or insufficient bioactivation, while **222b** was able to initially reduce parasitaemia at day 4 (82% suppression). However, the in vivo activity was reduced stepwise after 7 days (66%) and reached 50% of suppression after 14 days. 

The *α*-3,4-dichlorophenyl substitution of reverse fosmidomycin derivatives is a key structural element responsible for potent DXR inhibition and anti-infective in vitro activity [100]. Based on this successful modification, the *L*-alanine ethyl ester and *N*-acetyl *L*-tyrosine ethyl ester prodrugs (structures not shown) have been synthesized. Both derivatives lacked antiplasmodial activity, while the parent *α*-3,4-dichlorophenyl compound (**109**) exhibited nanomolar activity [37].

The Dowd group recently published arylalkyloxyamide analogs of **2** which potentially act as bisubstrate inhibitors for the natural substrate DXP and the NADPH cofactor [41]. Van Calenbergh and coworkers combined these findings with their phosphodiamidate prodrug strategy and developed prodrugs with improved penetration capabilities due to increased lipophilicity [183]. The promising whole-cell antimycobacterial activity of *L*-leucine ethyl ester phosphondiamidate (**221b**) was used as a starting point for further modification combined with different *N*-alkoxy residues (**223a**–**g**, Figure 41). All tested derivatives were less active against *Pf*-K1 (IC_50_ = 4.2–8.9 µM) compared to fosmidomycin (IC_50_ = 1.73 µM) and less active than **220f** against the nonvirulent *Mt* H37Ra strain. These derivatives were inactive against H37Rv *Mt*, but cytotoxicity against MRC-5 fibroblasts was significant.

In summary, the phosphobisamidate prodrug **220f** exhibited moderate in vitro activity against *Mt* H37Hv strains, whereas fosmidomycin was inactive, suggesting that this prodrug strategy may allow permeation through the highly lipophilic *Mt* cell wall. The combination of a phosphodiamidate prodrug and arylalkyloxyamide moieties (**223a**–**g**) was unsuccessful. In *Pf,* the tyrosine ester strategy was more promising than the phosphodiamidate strategy as *N*-acetyl- and *L*-tyrosine esters (**222a, b**) were the most active compounds. 

**Figure 42:**
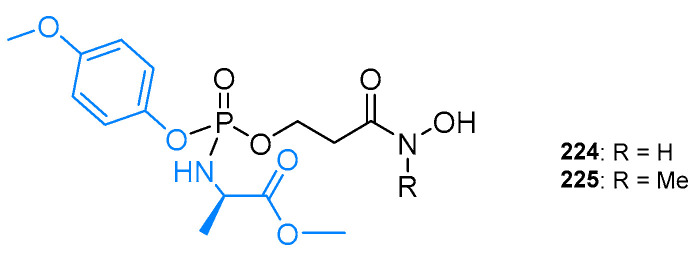
Structures of aryl phosphoramidate prodrugs of fosmidomycin (**224**) and FR900098 (**225**).

In 2019, Munier et al. synthesized aryl phosphoramidate prodrugs of fosfoxacin (**47**) and its *N*-methylated analog **48** bearing an *L*-alanine methyl ester and a 4-methoxyphenyl moiety (Figure 42) [184].

Both compounds (**224**–**225**) did not inhibit the growth of *E. coli* and *M. smegmatis* at the highest concentration of 400 nmol/disc in a paper disc diffusion assay. The authors demonstrated that **225** was not stable in the buffer used during the 48 h assay. Furthermore, the bioconversion into fosfoxacin was determined via incubation with carboxypeptidase Y (CPY), which catalyzes the hydrolyses of the carboxylic acid ester as the first step in bioactivation. While the results are not meaningful for **225** due to its instability in the buffer, **224** was completely converted to the amino acyl phosphoramidate intermediate with a half-life time of 20 h [184]. This new prodrug strategy for DXRi is interesting and should be used for phosphonic acid analogs, as this moiety is more stable compared to the phosphate moiety of the fosfoxacin analogs. 

In summary, the Van Calenbergh group successfully implemented the synthesis of a new prodrug type for DXR inhibitor discovery. However, significant in vivo activity was not achieved. To date, none of the applied prodrugs concepts presented in this chapter led to curative properties of the parent DXR inhibitors. However, the opportunities are not yet exhausted, and a combination with other concepts and the development of bisubstrate inhibitors may help accomplish the desired curative in vivo activity.

## 8. Fosmidomycin Conjugates and Hybrids

**Figure 43:**
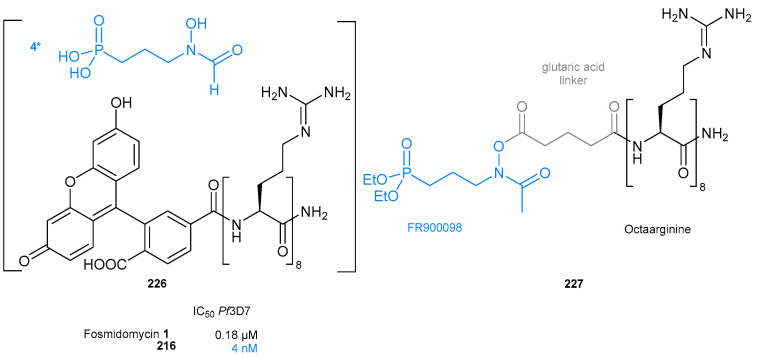
Structure and antiplasmodial activity of fosmidomycin (**226**) and FR900098 (**227**) octaarginine conjugates.

Sparr et al. addressed the poor permeability of fosmidomycin analogs by facilitating cellular uptake via a carrier. Cell-penetrating peptides (CPPs), e.g., polycationic oligoarginins are able to transport physiologically active compounds across membranes and act as a carrier or delivery vehicle [185,186,187,188]. The authors synthesized a salt of fosmidomycin and 6-carboxyfluorescein (FAM) labeled octaarginine amide in a 4-to-1 ratio (**226**, Figure 43). For the second target molecule, octaarginine was attached to the retro-hydroxamate group of diethyl phosphonate ester of FR900098 using a glutaric acid linker (**227**, Figure 43). First, the activity against asexual blood-stage *Pf*3D7 in comparison to fosmidomycin was determined. While the covalent conjugate **227** was less active than fosmidomycin, the salt **226** was 40-fold more active (IC_50_ = 4 nM). It was demonstrated that the FAM-octaarginine alone is no plasmodial growth inhibitor at concentrations up to 100 μM, suggesting the improved activity of **226** is caused by enhanced uptake rather than synergistic effects. In contrast, neither fosmidomycin nor salt **226** inhibited the growth of *T. gondii* (strain RH) in infected human foreskin fibroblasts (HFF). The authors demonstrated that this is due to the inability of both compounds to cross the parasite’s membranes. 

**Figure 44:**
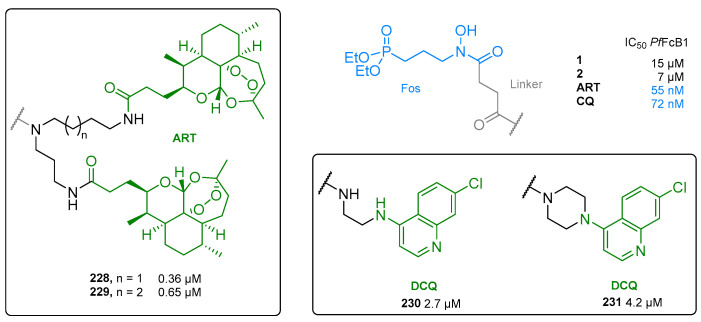
Antiplasmodial activities of artemisinin (ART)-conjugates **228**–**229** and desalkylchloroquin (DCQ) hybrids (**230**–**231**) against chloroquine-resistant *P. falciparum* FcB1 strain with ART and CQ as references.

It has been demonstrated that artemisinin–spermidine conjugates were up to 10-fold more active against the chloroquine-sensitive *Pf3*D7 strain [189]. This inspired Palla et al. to synthesize fosmidomycin conjugates and hybrids using the following fragments: the diethyl phosphonate ester of fosmidomycin (blue), a propyl carboxylic acid linker (grey) attached to the fosmidomycin hydroxamate nitrogen, a second linker (black), and the second pharmacophore (green). The artemisinin (ART) conjugates used the polyamines spermidine (**228**) or homospermidine (**229**) as a second linker, while the desalkylchloroquin (DCQ) hybrids were connected via an ethylenediamine (**230**) or piperazine (**231**) linker (Figure 44).

Compared to the diethyl phosphonate ester of fosmidomycin (structure not shown), which was completely inactive towards *P. falciparum* FcB1, the artemisinin conjugates **228** and **229** exhibited potent activity with IC_50_ values of 0.36 and 0.65 μM, respectively. However, these values are one order of magnitude higher than the IC_50_ value of artemisinin (IC_50_ = 55 nM). Compared to **228** and **229**, the DCQ hybrids **230** and **231** were active in the low micromolar range, but are still less active than the parent compound chloroquine. As demonstrated in this and previous studies, the phosphonic acid moiety of fosmidomycin is crucial for activity against DXR and its alkyl esters are not cleaved by plasma esterases. This suggests that the fosmidomycin pharmacophore of the reported conjugates is also inactive against DXR. Consequently, the potency of **228**–**231** in the conducted plasmodial growth assay is likely caused by the pharmacological effect of the second drug (ART, DCQ). 

To compensate for the poor physicochemical properties of fosmidomycin, the novel concepts of covalent and noncovalent attachment to drug delivery vehicles as well as drugs-conjugates and hybrid molecules have been demonstrated to be sufficient, but not well examined for DXR inhibitors. These first explorative studies were auspicious and provide opportunities for further improvement, e.g., application of more labile linker units for drug delivery vehicles for complete drug release or the use of DXR inhibitors bearing a free phosphonic acid moiety or proven ester prodrugs instead of stable dialkyl phosphonate esters. Furthermore, DXR assays must be conducted to elucidate if the inhibitory effect of the drug-drug and hybrid conjugates can be attributed to DXR inhibition and/or other target-based effects. 

## 9. DXR-Inhibitors Not Based on Fosmidomycin 

**Figure 45:**
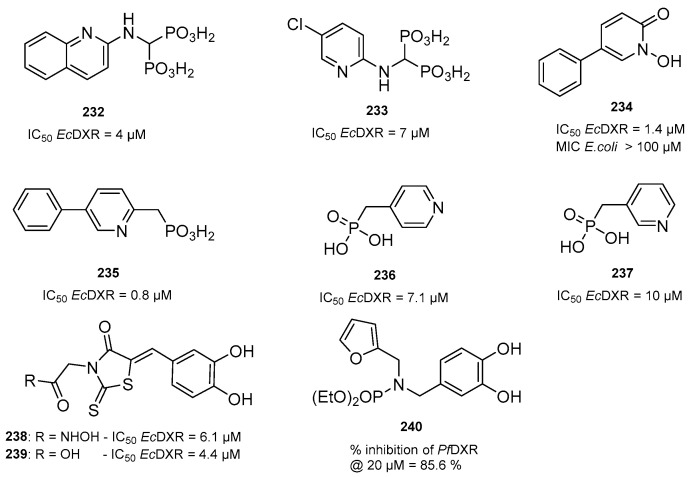
Structure and biological data of non-fosmidomycin-based DXR inhibitors **232**–**240**.

Non-fosmidomycin-based DXR inhibitors are herein defined as molecules that inhibit the isolated enzyme but do not follow the classical pharmacophore of fosmidomycin and reverse analogs. The number and success of these inhibitors have so far been limited. Due to the similarity of nitrogen-containing bisphosphonates to DMAPP and its antimalarial activity, Yajima et al. screened a library of bisphosphonic acid derivatives and identified compounds **232** and **233** as moderate DXR inhibitors with IC_50_ values of 4 and 7 μM, respectively (Figure 45). Interestingly, the crystal structures of **232** showed that the bisphosphonic acid group acts as the metal chelator and does not occupy the phosphonic acid pocket [190].

**Figure 46:**
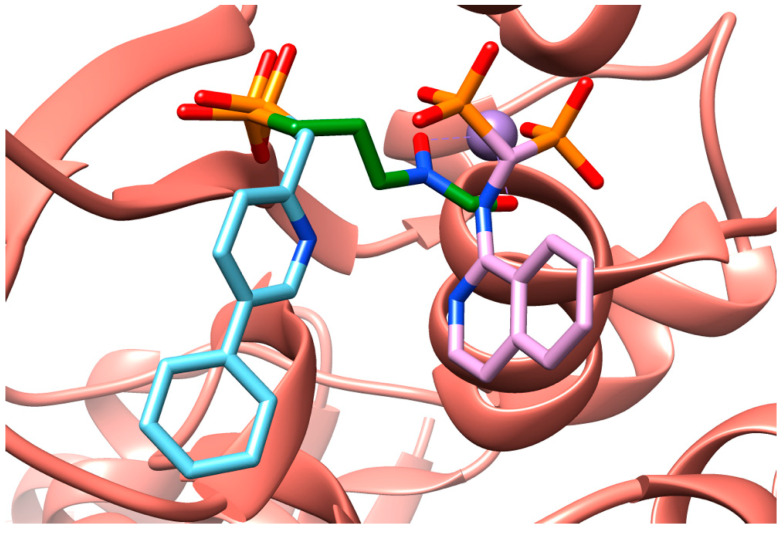
Superposition of fosmidomycin (green) PDB 1ONP, compound **235** (blue) PDB 3ANM and compound **232** (pink) PDB 1T1R in the structure of *Ec*DXR (salmon) PDB 1ONP.

Aiming to increase the lipophilicity of bisphosphonic acid-based inhibitor **232**, Deng et al. tested phenyl and benzyl derivatives containing different cyclic metal-binding moieties against *Ec*DXR, including inhibitor **234** (Figure 45). Among the tested compounds, 1-hydroxypyridin-2(*1H*)-one **234** inhibited *Ec*DXR at low micromolar concentrations (IC_50_ = 1.4 μM) [113]. To expand on the concept that an increase in lipophilicity is beneficial to the design of DXR inhibitors, the authors synthesized a series of arylphosphates containing electron-deficient aromatic moieties. Compounds containing pyridine rings (**236** and **237**, Figure 45) were active in micromolar concentrations, while compound **235** (Figure 45) inhibited the enzyme at submicromolar concentrations (**235** IC_50_ = 0.8 μM) [191]. The *Ec*DXR cocrystal structure demonstrated that the phosphonic acid group of **235** occupied the phosphate binding site of DXP and is not interacting with the catalytic metal (Figure 46).

To design a molecule that can interact with the catalytic metal ion and occupy the NADPH binding site, Zinglé et al. synthesized catechol-rhodanine-based DXR inhibitors. The compounds were found to be promiscuous inhibitors due to the formation of aggregates. With the inclusion of a detergent in the assay media, compounds **238** and **239** (Figure 45) showed inhibition at micromolar concentrations (**238** IC_50_ = 6.1 μM and **239** IC_50_ = 4.4 μM). Alteration of NADPH concentration in the assay did not alter the inhibition of **238** and **239**, suggesting that the compounds indeed did not interact with the NADPH recognition site [192].

More recently, in silico studies identified *N*-substituted phosphoramidate derivatives with a free phosphonic acid as potential DXR inhibitors. However, only the corresponding phosphonic acid esters were tested against the enzyme and compound **240** (Figure 45) was the most active compound of this series [193].

**Figure 47:**
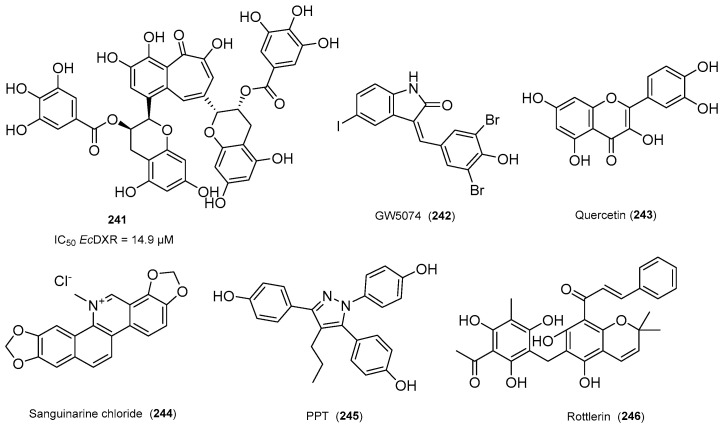
Structures of natural products (**241**–**246**) that potentially act as DXR inhibitors. Lead structures for the design of DXRi identified via HTS by Haymond et al. [195].

Theaflavins have been found to be non-competitive inhibitors of DXR. Theaflavin-3,3′-digallate (**241**, Figure 47) showed IC_50_ of 14.9 μM [194]. Docking studies suggested the compounds interact with the entrance of the substrate-binding site, supporting its non-orthosteric inhibition. A recent high throughput screening (HTS) campaign to identify inhibitors of the MEP pathway focusing on IspC and IspD used LOPAC (Library of Pharmacologically Active Compounds), a mixed library of 1280 commercial compounds, and 150 natural products [195]. The result of this work was the identification of novel chemical scaffolds as DXR inhibitors **241**–**246** shown in Figure 47. However, no experimental validation of these scaffolds has been conducted. 

## 10. Summary

The importance of the MEP pathway for the development of anti-infective drugs has been demonstrated by target- and cell-based assays in animal models and clinical trials with fosmidomycin. The majority of DXR inhibitor development focused on the structural optimization of the natural products fosmidomycin (**1**) and FR900098 (**2**). Both natural compounds potently inhibited the DXR enzymes of a panel of pathogenic bacteria and parasites such as *E. coli*, *M. tuberculosis,* and *P. falciparum*. 

Different design strategies have been employed to optimize the inhibitor profile and thereby pharmacokinetic (PK) and pharmacodynamic (PD) properties of **1** and **2**. The most promising analogs reported are *α*-phenyl and *α*-fluoro-substituted derivatives, which exhibited low nanomolar activity towards the DXR enzymes of *P. falciparum* and *M. tuberculosis*. However, this success was only in part transferred to whole-cell activity, and the in vivo efficacy of fosmidomycin has never been exceeded by an analog. The main shortcomings of **1** and **2** are their insufficient membrane permeability due to the charged phosphonic acid group and the short half-life as demonstrated by several unsatisfactory clinical trials. 

To enhance the permeability of **1**, **2**, and their analogs, which are present as phosphonate anions at physiological pH of 7.4, several phosphonate prodrug concepts were applied, including lipophilic ester and phosphonamidate prodrugs. Some mono and double prodrugs showed significantly improved antiparasitic and antibacterial activity compared to their parent compounds. However, even nanomolar in vitro activity could not be translated into potent or, at best, curative in vivo properties.

Recent results suggest that addressing the hydrophobic subpocket within the substrate binding site or the NADPH binding site (e.g., the adenosine-binding pocket) are promising strategies toward more lipophilic and druglike DXR inhibitors. However, so far, the postulated bisubstrate inhibitors have not been validated by co-crystal structures with DXR enzymes. Bisubstrate inhibitors are recent developments in the field of DXR inhibitors, and thus their potential may not have been fully explored. 

In more than 40 years of fosmidomycin drug research, no new highly active structure types have been developed based on numerous DXR crystal structures or discovered in drug screening approaches. A few examples of innovative drug design concepts have been used, including the conjugation of fosmidomycin to cell-penetrating-peptides (CPP) or fragments of other antimalarials. These concepts have so far not yielded improved anti-infectives, leaving room for further optimization. Since competitive catalytic site inhibitors of the phosphonohydroxamic acid type have not shown the expected in vivo efficacy, possible strategies include the development of small molecules with more druglike properties such as allosteric DXR inhibitors or DXR dimerization inhibitors. It remains to be seen whether the application of novel prodrug concepts from the field of antivirals will be more successful than the concepts used to date.

Despite the widespread distribution of the MEP pathway, significant antiparasitic activity has only been achieved against *Plasmodia*, but even here, no inhibitors with curative properties in animal models have been developed. Various studies revealed that the antiplasmodial properties of DXR inhibitors are more pronounced than their antibacterial effects, although several bacterial DXRs are inhibited with nanomolar IC_50_ values. Among the bacterial pathogens, studies with *M. tuberculosis* dominate, while studies with Gram-negative bacteria are underrepresented. To improve the antibacterial properties of fosmidomycin analogs, the design of siderophore conjugates to target resistant Gram-negative bacteria is another promising opportunity as demonstrated by the recently approved antibiotic Cefiderocol (Fetroja, 2020). 

In conclusion, since no further clinical studies with fosmidomycin as an antibiotic have been carried out since 1985, it cannot currently be assessed whether fosmidomycin could gain importance as a reserve antibiotic against certain bacteria. Current data provide hardly any arguments for the suitability of fosmidomycin for this purpose. Although approval for the treatment of Malaria could not be achieved so far, fosmidomycin in combination with approved antimalarials showed promising in vivo activity in humans with curative potential. On the other hand, the required repeat application of high doses of fosmidomycin and unimprovable cure rates as a standalone antimalarial are both unsatisfactory. As novel clinical studies with fosmidomycin in combination with clindamycin and artesunate are set to begin in 2022, fosmidomycin-based combination therapy for malaria is still possible. In our view, the DXR enzyme and the MEP pathway remain viable targets for anti-infective drug research, not only because of the vital importance of isoprenoids for the survival of pathogens, but also due to the pathway’s widespread prevalence and its absence in mammals.

## Data Availability

Data sharing not applicable.

## Supplementary Materials

The following supporting information can be downloaded at: https://www.mdpi.com/article/10.3390/ph15121553/s1. In this file, we describe obtained IC_50_ or MIC values of fosmidomycin (**1**) and FR900098 (**2**) for all tested microorganisms and the PDB codes from DXR crystal structures. Table S1: Antiparasitic and antibiotic data of fosmidomycin (**1**) and FR900098 (**2**) obtained from enzyme assays and growth inhibition assays; Table S2: Existing co-crystal structures of DXR enzymes.

## Abbreviations

ADME, absorption, distribution, metabolism, and excretion; BLAST, Basic Local Alignment Search Tool; DMAPP, dimethylallyl diphosphate; CPY, carboxypeptidase Y; DXP, 1-deoxy-D-xylulose-5-phosphate; DXR, 1-deoxy-D-xylulose-5-phosphate reductoisomerase; DXRi, 1-deoxy-D-xylulose-5-phosphate reductoisomerase inhibitor; Ec, *Escherichia coli*; *fsr*, fosmidomycin resistance gene; GlpT, glycerol-3-phosphate transporter; IPP, isopentenyl diphosphate; IspC, 1-deoxy-D-xylulose-5-phosphate reductoisomerase; IspD, 4-diphosphocytidyl-2C methyl-D-erythritol synthase; IspE, 4-diphosphocytidyl-2C-methyl-D-erythritol kinase; IspF, 2C-methyl-D-erythritol 2,4-cyclodiphosphate synthase; IspG, 2C-methyl-D-erythritol 2,4-cyclodiphosphate reductase; IspH, 1-hydroxy-2-methyl-2-(E)-butenyl-4-diphosphate reductase; HA, hydroxamic acid; HTS, high throughput screening; MBG, metal binding group; MDR, multidrug resistance; MEP, 2C-methyl-d-erythritol-4-phosphate pathway; MRC-5, Medical Research Council cell strain 5; MRSA, methicillin -resistant *Staphylococcus aureus*; MSSA, methicillin-sensitive *Staphylococcus aureus*; Mt, *Mycobacterium tuberculosis*; NHP, N-hydroxypyridone; n.i., no inhibition; NMP, non-mevalonate pathway; PDB, protein database; Pf, *Plasmodium falciparum*; Pf3D7, chloroquine-sensitive *plasmodium* falciparum strain 3D7; PfDd2, multiresistant strain *plasmodium falciparum* strain Dd2; Pf-K1, *Plasmodium falciparum* strain K1, a chloroquine- and pyrimethamine-resistant strain; PMB, p-methoxybenzyl; POC, isopropyloxycarbonyloxymethyl; POM, pivaloyloxymethyl; QSAR, quantitative structure–activity relationship; R, residue; SAR, structure–activity relationship; SCID, severe combined immunodeficiency; ssp., subspecies; THN, (5,6,7,8)-tetrahydronaphthaleneylmethyl; UhpT, hexose 6-phosphate transporter; Yp, *Yersinia pestis*.
