# Supplementary material for: Over 40 Years of Fosmidomycin Drug Research: A Comprehensive Review and Future Opportunities
Source: Pharmaceuticals (Basel). 2022 Dec 14;15(12):1553. doi: 10.3390/ph15121553 (PMC9782300; doi:10.3390/ph15121553)
Supplement: Supplementary file 1 [file pharmaceuticals-15-01553-s001.zip › pharmaceuticals-2028080-supplementary.pdf]

Over 40 years of fosmidomycin drug research: A comprehensive review and future opportunities

- Supporting information -

Talea Knak, Mona A. Abdullaziz, Stefan Höfmann, Leandro A. Alves Avelar, Saskia Klein, Matthew Martin, Adelbert Bacher, Markus Fischer, Nobutada Tanaka and Thomas Kurz

Table S1: Antiparasitic and antibiotic data of fosmidomycin (**1**) and FR900098 (**2**) obtained from enzyme assays and growth inhibition assays

	Fosmidomycin (**1**)		FR900098 (**2**)	
	DXR IC_{50}[a]	Whole-cell MIC[b]	DXR IC_{50}[a]	Whole-cell MIC[b]
Plasmodium falciparum Dd2	0.16 µM*[1]	IC_{50} = 0.81 µM[1]	0.015 µM*[1]	IC_{50} = 0.16 µM[1]
Plasmodium falciparum 3D7		IC_{50} = 0.88 µM[1]		IC_{50} = 0.16 µM[1]
Toxoplasma gondii	K_i = 0.090 µM*[2]	32% growth inhibition at 2.5 mM[3]	K_i = 0.048 µM*[2]	
Eimeria tenella		30% growth inhibition at 3.3 mM[3]		
Synechocystis sp.	K_i = 4 nM**[4]		K_i = 2 nM**[4]	
**Gram (+)**				
Bacillus anthracis		0.78 µg/ml[5]		50 µg/ml[5]
Bacillus subtilis (wild strain)		8 mM[6]		
Enterococcus faecalis		> 200 µg/ml[5]		> 200 µg/ml[5]
Mycobacterium tuberculosis	0.31 µM[7]	> 500 µg/ml[5]	2.91 µM[8]	> 500 µg/ml[5]
Staphylococcus aureus (MSSA)		> 200 µg/ml[5]		> 200 µg/ml[5]
S. aureus (MRSA)		200 µg/ml[5]		50 µg/ml[5]
Staphylococcus epidermidis		n.i.[10]		
Staphylococcus ludgenensis		n.i.[10]		
Staphylococcus pseudintermedius		0.5 – 1 µg/ml[10]		
Staphylococcus schleiferi		0.5 -8 µg/ml[10]		
**Gram (-)**				
Acinetobacter baumannii	46.8 nM[11]	> 512 µg/ml[11]	23.9 nM[11]	128 - > 512 µg/ml[11]
Burkholderia cepacia LMG 1222		> 512 µg/ml[12]		256 µg/ml[12]
Burkholderia multivorans LMG 13010		> 512 µg/ml[12]		256 µg/ml[12]
Burkholderia cenocepacia LMG 16656		> 512 µg/ml[12]		> 512 µg/ml[12]
E. coli K12	0.03 µM*[13]	> 12.5 µg/ml[5]	0.03 µM*[13]	200 µg/ml[5]
E.coli tolc		> 6.25 µg/ml[5]		12.5 µg/ml[5]
Francisella tularensis	247 nM[14]		230 nM[14]	
Francisella		136 µM[14]		254 µM[14]

novicida				
Pseudomonas aeruginosa	150 nM[9]		150 nM[9]	
Klebsiella pneumoniae	20.2 nM[11]	64 – 128 µg/ml[11]	23.1 nM[11]	256 µg/ml[11]
Yersinia pestis	710 nM[15]	128 µg/ml[16]	231 nM[15]	

[a] IC_{50} values for enzyme assays are given unless denoted otherwise
[b] MIC (minimum inhibitory concentration) values for whole cell assays are given unless denoted otherwise
* recombinant enzyme
** listed value obtained by preincubation studies
n.i. = no inhibition

Table S2: Existing co-crystal structures of DXR enzymes

Organism	PDB ID	Co-crystallized elements	Publication year
Mycobacterium tuberculosis	4OOF	NADPH, **1**, Mn^{2+}	2014
	3ZHZ	DXRi,	2013
	3ZI0	DXRi, Mn^{2+}	
	3ZHX	DXRi, Mn^{2+}	
	3ZHY	DXRi, NADPH, Mn^{2+}	
	2Y1C	Mn^{2+}	2011
	2Y1D	DXRi, Mn^{2+}	
	2Y1E	Mn^{2+}	
	2Y1F	NADPH, DXRi, Mn^{2+}	
	2Y1G	DXR, Mn^{2+}	
	2JCV	NADPH, **1**	2007
	2JCX	NADPH, **1**	
	2JCY	None	
	2JD0	NADPH	
	2JD1	NADPH, Mn^{2+}	
	2JD2	Mn^{2+}	
	2C82	None	2006
	4AIC	NADPH, **1**, Mn^{2+}	2012
	4RCV	NADPH, Mn^{2+}	2015
	4A03	NADPH, **2**, Mn^{2+}	2012
	3RAS	NADPH, DXRi, Mn^{2+}	2011
Plasmodium falciparum	3AU8	Mn^{2+}, NADPH	2011
	3AU9	Mg^{2+}, NADPH, **1**	
	3AUA	Mg^{2+}, NADPH, **2**	
	4Y67	DXRi, Mn^{2+}	2015
	4Y6S	DXRi, Mn^{2+}	
	4Y6R	DXRi, Mn^{2+}	
	4Y6P	DXRi, Mn^{2+}	
	3WQS	NADPH, DXRi, Mg^{2+}	2014
	3WQR	NADPH, DXRi, Mg^{2+}	
	3WQQ	NADPH, DXRi, $Mg2^{+}$	
	5JMP	DXRi, Mn^{2+}	2016
	5JO0	DXRi, Mn^{2+}	
	5JNL	DXRi, Mn^{2+}	
	5JBI	DXRi, Mn^{2+}	
	5JC1	DXRi, Mn^{2+}	
	5JAZ	DXRi, Mn^{2+}	
	5JMW	DXRi, Mn^{2+}	
	4QJB	Mg^{2+}	2014
	4GAE	DXRi, NADPH, Mn^{2+}	2013
	4KP7	DXRi, NADPH, Mn^{2+}	2013
	4QOX	Mg^{2+}, DXRi	2014

Organism	PDB ID	Co-crystallized elements	Publication year
Escherichia coli	2EGH	NADPH, **1**, Mg^{+2}	2007
	3ANL	NADPH, DXRi	2011
	3ANM	NADPH, DXRi	
	3ANN	NADPH, DXRi	
	1Q0L	NADPH, **1**	2004
	1Q0Q	NADPH, DXP	
	1Q0H	NADPH, **1**	
	1T1R	DXRi	2004
	1T1S	DXRi, Mg^{2+}	
	1K5H	None	2002
	3R0I	DXRi, Mn^{2+}	2011
	1JVS	NADPH	2002
Zymomonas mobilis	1R0K	None	2004
	1R0L	NADPH	
Thermotoga maritima	3A06	NADPH, **1**, Mg^{2+}	2010
	3A14	NADPH, Mg^{2+}	
Yersinia pestis	3IIE	Mg^{2+}	2009
	5DUL	NADPH	2015
Staphylococcus schleiferi	6MH4	None	2020
	6MH5	**1**	
Brucella abortus	3UPY	**1**, Mg^{2+}	2012
	3UPL	Mg^{2+}	
Acinetobacter baumannii	4ZN6	None	2015
	7S04	NADPH, Mg^{2+}, **2**	2021
Moraxella catarrhalis	4ZQE	None	2016
	4ZQF	Mg^{2+}, **1**	
	4ZQH	NADPH, Mg^{2+}, **1**	
	4ZQG	NADPH, Mg^{2+}, **1**	
Vibrio vulnificus	5KRR	Mn^{2+}	2017
	5KS1	Mn^{2+}	
	5KRV	Arginine	
	5KRY	None	
	5KQO	None	

References

(1) Brücher, K.; Gräwert, T.; Konzuch, S.; Held, J.; Lienau, C.; Behrendt, C.; Illarionov, B.; Maes, L.; Bacher, A.; Wittlin, S.; et al. Prodrugs of Reverse Fosmidomycin Analogues. *Journal of Medicinal Chemistry* **2015**, *58* (4), 2025-2035. DOI: 10.1021/jm5019719.

(2) Cai, G.; Deng, L.; Xue, J.; Moreno, S. N. J.; Striepen, B.; Song, Y. Expression, characterization and inhibition of Toxoplasma gondii 1-deoxy-d-xylulose-5-phosphate reductoisomerase. *Bioorganic & Medicinal Chemistry Letters* **2013**, *23* (7), 2158-2161. DOI: https://doi.org/10.1016/j.bmcl.2013.01.097.

(3) Clastre, M.; Goubard, A.; Prel, A.; Mincheva, Z.; Viaud-Massuart, M. C.; Bout, D.; Rideau, M.; Velge-Roussel, F.; Laurent, F. The methylerythritol phosphate pathway for isoprenoid biosynthesis in coccidia: presence and sensitivity to fosmidomycin. *Exp Parasitol* **2007**, *116* (4), 375-384. DOI: 10.1016/j.exppara.2007.02.002 From NLM.

(4) Woo, Y.-H.; Fernandes, R. P. M.; Proteau, P. J. Evaluation of fosmidomycin analogs as inhibitors of the Synechocystis sp. PCC6803 1-deoxy-d-xylulose 5-phosphate reductoisomerase. *Bioorganic & Medicinal Chemistry* **2006**, *14* (7), 2375-2385. DOI: https://doi.org/10.1016/j.bmc.2005.11.012.

(5) Uh, E.; Jackson, E. R.; San Jose, G.; Maddox, M.; Lee, R. E.; Lee, R. E.; Boshoff, H. I.; Dowd, C. S. Antibacterial and antitubercular activity of fosmidomycin, FR900098, and their lipophilic analogs. *Bioorg Med Chem Lett* **2011**, *21* (23), 6973-6976. DOI: 10.1016/j.bmcl.2011.09.123 PubMed.

(6) Sivy, T. L.; Fall, R.; Rosenstiel, T. N. Evidence of isoprenoid precursor toxicity in Bacillus subtilis. *Biosci Biotechnol Biochem* **2011**, *75* (12), 2376-2383. DOI: 10.1271/bbb.110572 From NLM.

(7) Dhiman, R. K.; Schaeffer, M. L.; Bailey, A. M.; Testa, C. A.; Scherman, H.; Crick, D. C. 1-Deoxy-D-Xylulose 5-Phosphate Reductoisomerase (IspC) from *Mycobacterium tuberculosis*: towards Understanding Mycobacterial Resistance to Fosmidomycin. *Journal of Bacteriology* **2005**, *187* (24), 8395-8402. DOI: doi:10.1128/JB.187.24.8395-8402.2005.

(8) San Jose, G.; Jackson, E. R.; Haymond, A.; Johny, C.; Edwards, R. L.; Wang, X.; Brothers, R. C.; Edelstein, E. K.; Odom, A. R.; Boshoff, H. I.; et al. Structure–Activity Relationships of the MEPicides: N-Acyl and O-Linked Analogs of FR900098 as Inhibitors of Dxr from Mycobacterium tuberculosis and Yersinia pestis. *ACS Infectious Diseases* **2016**, *2* (12), 923-935. DOI: 10.1021/acsinfecdis.6b00125.

(9) Altincicek, B.; Hintz, M.; Sanderbrand, S.; Wiesner, J.; Beck, E.; Jomaa, H. Tools for discovery of inhibitors of the 1-deoxy-D-xylulose 5-phosphate (DXP) synthase and DXP reductoisomerase: an approach with enzymes from the pathogenic bacterium Pseudomonas aeruginosa. *FEMS Microbiology Letters* **2000**, *190* (2), 329-333. DOI: 10.1111/j.1574-6968.2000.tb09307.x (acccessed 1/10/2022).

(10) Misic, A. M.; Cain, C. L.; Morris, D. O.; Rankin, S. C.; Beiting, D. P.; Fey, P. D. Divergent Isoprenoid Biosynthesis Pathways in *Staphylococcus* Species Constitute a Drug Target for Treating Infections in Companion Animals. *mSphere* **2016**, *1* (5), e00258-00216. DOI: doi:10.1128/mSphere.00258-16.

(11) Ball, H. S.; Girma, M. B.; Zainab, M.; Soojhawon, I.; Couch, R. D.; Noble, S. M. Characterization and Inhibition of 1-Deoxy-d-Xylulose 5-Phosphate Reductoisomerase: A Promising Drug Target in Acinetobacter baumannii and Klebsiella pneumoniae. *ACS Infectious Diseases* **2021**, *7* (11), 2987-2998. DOI: 10.1021/acsinfecdis.1c00132.

(12) Messiaen, A. S.; Verbrugghen, T.; Declerck, C.; Ortmann, R.; Schlitzer, M.; Nelis, H.; Van Calenbergh, S.; Coenye, T. Resistance of the Burkholderia cepacia complex to fosmidomycin and fosmidomycin derivatives. *Int J Antimicrob Agents* **2011**, *38* (3), 261-264. DOI: 10.1016/j.ijantimicag.2011.04.020 From NLM.

(13) Haemers, T.; Wiesner, J.; Poecke, S. V.; Goeman, J.; Henschker, D.; Beck, E.; Jomaa, H.; Calenbergh, S. V. Synthesis of α-substituted fosmidomycin analogues as highly potent Plasmodium falciparum growth inhibitors. *Bioorganic & Medicinal Chemistry Letters* **2006**, *16* (7), 1888-1891. DOI: https://doi.org/10.1016/j.bmcl.2005.12.082.

(14) McKenney, E. S.; Sargent, M.; Khan, H.; Uh, E.; Jackson, E. R.; Jose, G. S.; Couch, R. D.; Dowd, C. S.; van Hoek, M. L. Lipophilic Prodrugs of FR900098 Are Antimicrobial against Francisella novicida In Vivo and In Vitro and Show GlpT Independent Efficacy. *PLOS ONE* **2012**, *7* (10), e38167. DOI: 10.1371/journal.pone.0038167.

(15) Haymond, A.; Johny, C.; Dowdy, T.; Schweibenz, B.; Villarroel, K.; Young, R.; Mantooth, C. J.; Patel, T.; Bases, J.; San Jose, G.; et al. Kinetic characterization and allosteric inhibition of the Yersinia pestis 1-deoxy-D-xylulose 5-phosphate reductoisomerase (MEP synthase). *PLoS One* **2014**, *9* (8), e106243. DOI: 10.1371/journal.pone.0106243 From NLM.

(16) Ball, H. S.; Girma, M.; Zainab, M.; Riley, H.; Behrendt, C. T.; Lienau, C.; Konzuch, S.; Avelar, L. A. A.; Lungerich, B.; Soojhawon, I.; et al. Inhibition of the Yersinia pestis Methylerythritol Phosphate Pathway of Isoprenoid Biosynthesis by α-Phenyl-Substituted Reverse Fosmidomycin Analogues. *ACS Omega* **2020**, *5* (10), 5170-5175. DOI: 10.1021/acsomega.9b04171.
